# Effect of Sodium Benzoate vs Placebo Among Individuals With Early Psychosis: A Randomized Clinical Trial

**DOI:** 10.1001/jamanetworkopen.2020.24335

**Published:** 2020-11-10

**Authors:** James G. Scott, Andrea Baker, Carmen C. W. Lim, Sharon Foley, Frances Dark, Anne Gordon, David Ward, Drew Richardson, George Bruxner, K. Martin Beckmann, Sean Hatherill, Stephen Stathis, Krystal Dixon, Alexander E. Ryan, Brett C. McWhinney, Jacobus P. J. Ungerer, Michael Berk, Olivia M. Dean, Sukanta Saha, John McGrath

**Affiliations:** 1 Queensland Centre for Mental Health Research, The Park Centre for Mental Health, Wacol, Australia; 2 QIMR Berghofer Medical Research Institute, Herston, Australia; 3 Metro North Mental Health Service, Herston, Australia; 4 Queensland Brain Institute, University of Queensland, St Lucia, Australia; 5 Emotional Health Unit, Mater Hospital, South Brisbane, Australia; 6 Metro South Mental Health, MacGregor, Australia; 7 Metro North Mental Health, Royal Brisbane and Women’s Hospital, Herston, Australia; 8 West Moreton Mental Health Service, Ipswich, Australia; 9 Metro North Mental Health, Caboolture and Redcliffe Hospitals, Caboolture, Australia; 10 School of Medicine, Logan Hospital, Griffith University, Meadowbrook, Australia; 11 Child and Youth Mental Health Service, Metro South Mental Health, Logan Hospital, Meadowbrook, Australia; 12 Metro South Addiction and Mental Health Services, Logan Hospital, Meadowbrook, Australia; 13 Queensland Children’s Hospital, South Brisbane, Australia; 14 Child Health Research Centre, University of Queensland, Brisbane, Australia; 15 Mental Health and Addiction Services, Sunshine Coast Hospital and Health Service, Birtinya, Australia; 16 School of Medicine, Sunshine Coast University Hospital, Griffith University, Birtinya, Australia; 17 University of Queensland Centre for Clinical Research, Herston, Australia; 18 Department of Chemical Pathology, Pathology Queensland, Royal Brisbane and Women’s Hospital, Herston, Australia; 19 Department of Chemical Pathology, Pathology Queensland, Brisbane, Australia; 20 School of Biomedical Sciences, University of Queensland, St Lucia, Australia; 21 Institute for Mental and Physical Health and Clinical Translation, School of Medicine, Barwon Health, Deakin University, Geelong, Australia; 22 National Centre for Register-based Research, Department of Economics and Business Economics, Aarhus University, Aarhus, Denmark

## Abstract

**Importance:**

There is evidence that sodium benzoate (BZ) may be an effective adjunctive treatment for schizophrenia. The clinical efficacy of BZ has been investigated in chronic schizophrenia; however, the efficacy of this agent has not been studied in individuals with early psychosis.

**Objective:**

To examine the clinical efficacy of the adjunctive use of BZ for symptoms in people with early psychosis.

**Design, Setting, and Participants:**

Using a placebo-controlled double-masked parallel-group design, this randomized clinical trial was conducted from August 2015 to July 2018. Participants aged between 15 and 45 years experiencing early psychosis were enrolled from 5 major clinical sites in Queensland, Australia. Data analysis was conducted from October 2018 to February 2020.

**Interventions:**

Participants were randomized 1:1 (50 participants in each group) to receive 500 mg of sodium benzoate twice daily or placebo for 12 weeks.

**Main Outcomes and Measures:**

The primary efficacy outcome was the Positive and Negative Syndrome Scale (PANSS) total score at 12 weeks. The key secondary efficacy measures were (1) the Clinical Global Impression score, (2) the Hamilton Depression Rating Scale for depression, (3) functioning as assessed by the clinician-rated Global Assessment of Function, and (4) the Assessment of Quality of Life Scale. The PANSS subscale scores and impact on selected amino acid concentrations were also assessed.

**Results:**

The study comprised 100 participants with a mean (SD) age of 21.4 (4.1) years, of whom 73 (73%) were male individuals. The mean (SD) baseline PANSS score was 75.3 (15.4). We found no improvement in total PANSS score in the BZ group compared with the placebo group. The end result of least-squares mean difference (SE) for total PANSS was −1.2 (2.4) (*P* = .63). There were no differences in any subscales of the PANSS, any secondary measures, nor any amino acid concentrations. The dose of BZ was well tolerated without any clinically significant treatment-emergent adverse event differences between BZ and placebo groups.

**Conclusions and Relevance:**

In this randomized clinical trial, there was no evidence that adjunctive use of 500 mg of BZ twice daily is an effective treatment for individuals with early psychosis.

**Trial Registration:**

anzctr.org.au Identifier: ACTRN12615000187549

## Key Points

**Question:**

Is 1000 mg of sodium benzoate daily an effective adjunctive treatment for early psychosis?

**Findings:**

In this randomized clinical trial of 100 participants with early psychosis, there was no significant improvement in the total Positive and Negative Syndrome Scale (PANSS) score for those who received 1000 mg of sodium benzoate for 12 weeks compared with those who received placebo. There were no end point differences in any subscales of the PANSS, any secondary clinical measures, nor any blood amino acid concentrations between treatment and placebo groups.

**Meaning:**

In this study, there was no evidence that adjunctive use of 1000 mg of sodium benzoate daily is an effective treatment for individuals with early psychosis.

## Teaser

This randomized clinical trial examines the clinical efficacy of the adjunctive use of sodium benzoate for symptoms in people with early psychosis.

## Introduction

People living with psychosis all too commonly experience symptoms that are refractory to treatment, which contributes to significant disability,^1^ resulting in a high disease burden.^2^ Treatment guidelines for psychosis recommend antipsychotic therapy accompanied by psychosocial interventions.^3^ While antipsychotic medications modulating dopaminergic activity are generally effective in reducing positive symptoms, many people continue to experience persistent impairment. It is now recognized that the pathophysiology underlying psychosis extends beyond dopaminergic dysregulation. Hypofunction of the N-methyl-D-aspartate (NMDA) receptors^4^ is another proposed mechanism that is not addressed by standard antipsychotic medications. Trials of pharmacological agents that act on molecular targets such as the NMDA receptors may allow us to expand psychosis treatments.^5^

NMDA receptors consist of 2 main subunits, the glutamate and glycine binding sites. Options for enhancing NMDA function are limited to modulators of the glycine binding site, as increasing glutamate levels can lead to excitotoxicity of neural cells. D-amino acids (DAAs) such as D-serine and D-alanine are agonists of the glycine subunit and have shown promise as adjunctive therapies for the treatment of schizophrenia.^6^ However, the oxidation of DAAs by the flavoenzyme D-amino acid oxidase (DAAO) limits their use in treatment.^7,8^ Oxidation reduces their bioavailability and increases products that are potentially nephrotoxic in high dosages.^9,10^

There has been interest in the use of compounds that inhibit DAAO, which would lead to increased concentration of DAA. Sodium benzoate (BZ), which is not related to the widely used benzodiazepine class of medications, is a widely used food preservative and an inhibitor of DAAO. There is evidence from recent clinical trials that BZ has efficacy and is tolerable in people with schizophrenia,^11,12^ including a study of 52 participants with schizophrenia (mean duration of illness, 14.6 years) randomized to adjunctive BZ (1000 mg per day) or placebo, in addition to usual treatment for a period of 6 weeks.^13^ At the end of the study, all domains of the PANSS had improved by a mean of 21% (effect size range, 1.16-1.69), and improvements were also found on selected measures of neurocognition. A second study focused on 60 participants with treatment-resistant schizophrenia who were taking clozapine.^14^ Participants were randomized to 1 g/d of BZ, 2 g/d of BZ, or placebo. Those receiving BZ (1 or 2 g/d) had significant improvements in negative symptoms as measured by the Scale for the Assessment of Negative Symptoms. Additionally, the group who received 2 g/d of BZ had large and significant reductions in total PANSS score and increases on the Quality of Life Scale. Both studies found that BZ had a favorable safety profile.

Together these trials suggest that BZ may be an effective and well-tolerated adjunctive therapy for those with schizophrenia. However, both studies recruited participants with long-standing and/or treatment-resistant schizophrenia. Considerable recent attention has focused on optimizing treatments during the early phases of psychosis.^15,16^ Assertive treatments for those with early psychosis may result in better outcomes with greater symptom reduction and improved functioning compared with the same treatments when given to individuals with long-standing psychotic disorders. To our knowledge, no studies to date have examined the efficacy of BZ as a treatment for those with early psychosis. Therefore, the main aim of this study (Cadence BZ) was to investigate the impact of adjunctive BZ on symptom, functional, and quality-of-life outcomes in participants with early psychosis. The hypotheses were that compared with placebo, people with early psychosis treated with 1000 mg/d of adjunctive BZ for 12 weeks would experience a significant reduction in symptoms of psychosis as measured by the PANSS. In addition, we examined whether adjunctive BZ would alter levels of the peripheral amino acids D-alanine and L-alanine as well as D-serine and L- serine in peripheral blood.

## Methods

### Study Design

Cadence BZ was a multicenter, randomized clinical trial of either BZ at 1 g/d or placebo, adjunctive to receiving usual clinical care within public mental health early psychosis and other services. The study was approved by the Metro South Human Research Ethics Committee and registered with the Australian New Zealand Clinical Trials Registry. The protocol and the statistical analysis plan have been previously published^17,18^ (Supplement 1). All participants provided written informed consent. The trial was conducted from August 2015 to July 2018. Data analysis and reporting was done in accordance to the Consolidated Standards of Reporting Trials (CONSORT) reporting guideline.^23^

Help-seeking individuals were eligible to participate if they were aged between 15 and 45 years, met diagnostic criteria for a *Diagnostic and Statistical Manual of Mental Disorders* (Fourth Edition) psychotic disorder (schizophrenia, schizophreniform psychosis, delusional disorder, bipolar disorder, psychosis not otherwise specified) with illness onset within the last 2 years, had received antipsychotic medications for at least 1 continuous month within the 2-year period of illness, and had a PANSS total score of at least 55. Diagnosis was confirmed with the Diagnostic Interview for Psychosis.^19^ Participants were eligible to participate if they had the capacity to consent and were able to follow the study instructions and procedures. Exclusion criteria were known allergies to BZ or to food preservatives in general; comorbid physical illnesses requiring additional treatments or hospitalizations; inability to communicate in English; currently pregnant, lactating or planning to become pregnant during the study period; concomitant participation in another clinical trial; and inability to understand and follow the study instructions and procedures.

### Study Interventions

Participants received either 500 mg of BZ twice daily (1000 mg/d total) or microcrystalline cellulose in matched gelatin capsule (placebo) for 12 weeks. This dose was selected based on the original study of BZ in people with chronic schizophrenia,^13^ in which a significant reduction in symptoms compared with placebo without significant adverse events was reported. Participants were being treated as clinically indicated by early psychosis services or community care teams in 1 of 5 public mental health services in southeast Queensland. Participants were clinically assessed at baseline and weeks 2, 4, 6, 8, 10, and 12 and received an AUD $40 (US $28.34) retail shop gift card at the end of weeks 2, 6, and 12 (total reimbursement, AUD $120 [US $85.01]).

The investigational products were manufactured in accordance with current Good Manufacturing Practice in a Therapeutic Goods Administration–licensed facility. Participants returned all unused study medication (ie, unopened blister packs or capsules not taken) and empty blister packs to research personnel. All unused supplies of study medication were accounted for and documented by the designated research pharmacist. Treatment adherence was determined by calculating the mean number of pills taken per day across the 12 week study and dividing this by 2 (the daily protocol dosage). Those who had taken more than 80% of capsules were assessed as adherent.

### Randomization and Blinding

Participants were randomized in a 1:1 ratio using a computer-generated randomization table, stratified by the 5 sites. A separate randomization table was generated for each site, and each list ensured randomization occurred in blocked groups of 4. The allocation sequence was generated by an independent statistician, and the dispensing of study medication was supervised by an independent research pharmacist. All participants, clinical teams, investigators, and the study statistician were masked to treatment assignment for the duration of the study.

### Outcome Measures

The primary outcome measure was total score on the PANSS at 12 weeks. Secondary measures included the PANSS subscale scores, the Hamilton Depression Rating Scale–17 items (HDRS) for depression, functioning as assessed by the clinician rated Global Assessment of Function (GAF), the Clinical Global Impression Scale (CGI), and health-related quality of life measured by the 12-item Assessment of Quality of Life Scale (AQoL).

A 25-mL sample of whole blood was collected at baseline and end point to measure the concentration of amino acids oxidized by DAAO, the action of which is inhibited by BZ. These were D-alanine and L-alanine as well as D-serine and L-serine. Participants who declined providing blood samples were still able to participate in the trial. The method used to measure concentrations was a modification of the high-performance liquid chromatography technique described by Hashimoto et al.^20^

### Adverse Events

Research personnel monitored each participant for adverse events (AEs) continuously through the study, and participants were encouraged to spontaneously report any unusual events, feelings, or sensations. Each AE was monitored until resolution or to the end of the 12-week protocol.

### Statistical Analysis

Using a 2-tailed test with α set at .05 and 90% power to detect a minimum clinical meaningful difference in total PANSS score of at least 5 units (SD, 14.3) between treatment groups, a sample of 39 persons in each group was needed. With a predicted attrition of 20% over the 12-week study, the recruitment target was 100 participants.

The analysis performed was based on intention-to-treat that included all 100 randomized participants in the analysis. A per-protocol analysis was also conducted that included participants (1) who stayed on the trial for 12 weeks, (2) who were treatment adherent (ie, adherence of >80%), and (3) who had absence of other major protocol violations. Missing values were handled by using a mixed-effects model repeated measures (MMRM) analysis. This is as robust in maintaining statistical properties of a test compared with the multiple imputation method in handling missing values,^21^ assuming that participants dropping out from the study are unrelated to outcome conditioned on the study covariates (ie, missing at random assumption).

Participants were compared at baseline on the outcome measures and on sex, age, living situation, waist circumference, height, blood pressure, and body mass index (BMI; calculated as weight in kilograms divided by height in meters squared). Tests of significance were computed comparing the intervention and placebo groups for each baseline characteristic. Baseline characteristics were included as covariates in the multivariable analysis to account for any imbalance that may have occurred by chance between the groups.

Differences between the intervention group and the placebo group in the total PANSS scores were analyzed using the MMRM.^22^ We included treatment group, week, and treatment-week interaction as fixed effects and intercept as random effects in the model. We used the first order autoregressive as the covariance structure of the MMRM model. MMRM models were used to examine differences in secondary outcome measures of the PANSS subscales, CGI, GAF, HDRS, and the AQoL. Bonferroni corrections were used to adjust the *P* value threshold for the 7 symptom-related secondary outcomes (α = .05 / 7 = .007). For the assessment of amino acid concentration, we used an analysis of covariance model to evaluate whether the mean end point values were equal across the BZ and placebo groups while controlling for the baseline concentrations. All analyses were carried out using the SAS software version 9.4 (SAS Institute).

## Results

### Participants

**Figure 1.**
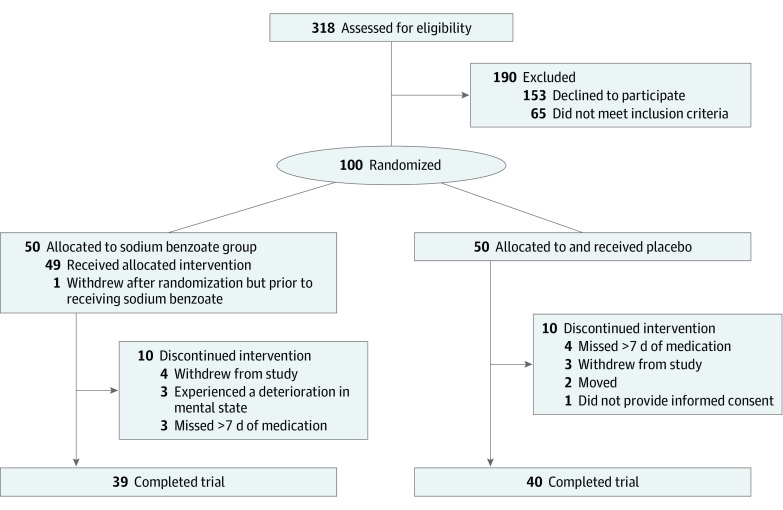
Study Flow Diagram

The study comprised 100 participants with mean (SD) age of 21.4 (4.1) years, of whom 73 (73%) were male individuals. These participants were recruited from 318 individuals who were screened for eligibility. Of these, 153 (48.1%) declined participation, and 65 (20.4%) did not fulfil eligibility criteria (Figure 1). One participant who was randomized to the BZ group discontinued prior to receiving the intervention without providing any data; therefore, the intention-to-treat analysis included 99 participants. During the study, 10 participants from each group dropped out.

Of the 99 participants who received treatment, 83 (84%) had schizophrenia. The remaining 16 (16%) had affective psychoses (eg, bipolar affective disorder, schizoaffective disorder). Within this group, 7 (44%) received BZ and 9 (56%) received placebo. Baseline characteristics were similar in the BZ and placebo groups, except for mean (SD) waist circumference, which was higher in the placebo group than the BZ group (98.4 [15.6] cm vs 92.0 [11.7] cm) (Table 1 and Table 2). The mean (SD) BMI of the entire cohort was 26.8 (6.2), and the mean (SD) waist circumference was 95.3 (14.1) cm. Most participants (43 [88%]) lived independently. The mean (SD) baseline PANSS score was 75.3 (15.4). At baseline, 83 patients (83%) were taking antipsychotics alone, 13 (13%) were taking both antipsychotics and mood stabilizers, and 4 (4%) were taking mood stabilizers alone. The 2 most commonly used antipsychotics were olanzapine and aripiprazole. Medications prescribed in the 2 groups at baseline are shown in eTable 1 and eTable 2 in Supplement 2.

**Table 1.** Baseline Age and Anthropometric Characteristics and Primary and Secondary Measures

Characteristics	Mean (SD)		
	Sodium benzoate group (n = 49)	Placebo group (n = 50)	Total (N = 99)
Age, y	21.7 (4.7)	21.2 (3.4)	21.4 (4.1)
Waist circumference, cm	92.0 (11.7)	98.4 (15.6)	95.3 (14.1)
Height, cm	175.8 (10.2)	174.9 (10.7)	176.7 (9.8)
Weight, kg	80.5 (18.1)	85.5 (24.2)	83.0 (21.5)
BMI	25.8 (5.3)	27.7 (6.8)	26.8 (6.2)
Total PANSS score	75.9 (16.3)	74.7 (14.7)	75.3 (15.4)
PANSS positive score	19.1 (6.1)	17.6 (5.9)	18.4 (6)
PANSS negative score	18.2 (5.9)	19.1 (6.8)	18.6 (6.3)
PANSS general psychopathology score	38.6 (8.3)	38 (7.3)	38.3 (7.8)
Clinical Global Impression score	4.3 (0.9)	4.1 (0.7)	4.2 (0.8)
Global Assessment of Functioning score	52.7 (9.4)	52 (9.3)	52.4 (9.3)
Hamilton Depression Rating Scale score	10.2 (5.6)	8.7 (5.9)	9.4 (5.8)
Assessment of Quality of Life score	19.6 (4)	18.8 (3.4)	19.2 (3.7)

Abbreviations: BMI, body mass index (calculated as weight in kilograms divided by height in meters squared); PANSS, Positive and Negative Syndrome Scale.

**Table 2.** Baseline Sex and Living Condition Characteristics

Characteristic	Patients, No. (%)		
	Sodium benzoate group (n = 49)	Placebo group (n = 50)	Total (N = 99)
Male participants	38 (77.6)	35 (70.0)	73 (73.7)
Living conditions			
Hospital inpatients	5 (10.2)	4 (8.0)	9 (9.1)
Supported housing	1 (2.0)	2 (4.0)	3 (3.0)
Independent	43 (87.8)	44 (88.0)	87 (87.9)

### Efficacy

**Figure 2.**
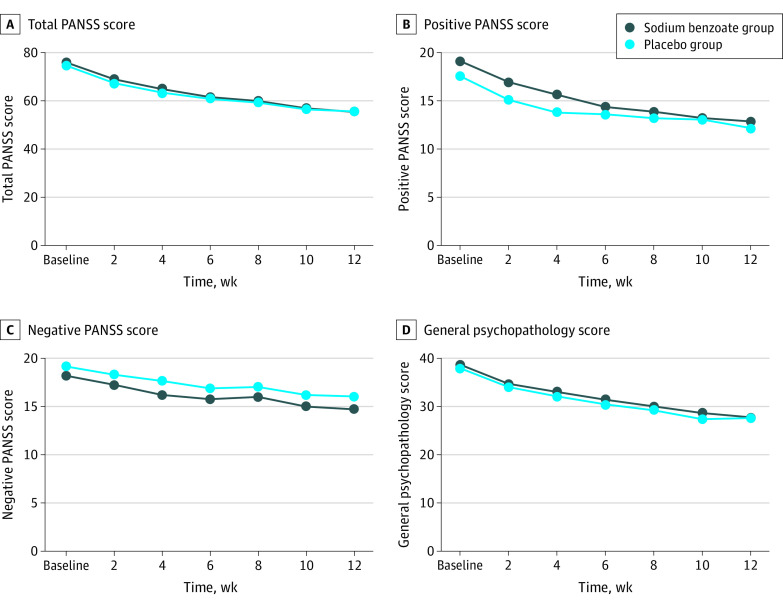
Total Positive and Negative Syndrome Scale (PANSS) and PANSS Subscale Scores After 12 Weeks in the Treatment and Placebo Groups

Both groups showed a reduction in PANSS total scores over time. After 12 weeks of treatment with 1000 mg of BZ per day, there was no significant difference in PANSS total score in the treatment (BZ) vs placebo groups (Table 3 and Figure 2). The end point least-square mean difference (SE) of the total PANSS score between BZ and placebo was −1.2 (2.4), which was not statistically significant (*t* = −0.49, *P* = .63) (Table 3).

**Table 3.** Efficacy Measure of PANSS and Other Scales After 12 Weeks of BZ Treatment

Scale	Mean (SE)							Significance test	
	Treatment period least-square						Difference in score, placebo vs BZ		
	Week 2	Week 4	Week 6	Week 8	Week 10	Final visit		*t*	*P* value
**Primary measures**									
PANSS total score									
Placebo	67.0 (1.6)	63.5 (1.6)	61.1 (1.7)	59.6 (1.7)	56.9 (1.7)	56.0 (1.8)	–1.2 (2.4)	–0.49	.63
BZ	67.4 (1.6)	64.8 (1.7)	62.8 (1.7)	61.4 (1.7)	58.9 (1.7)	57.2 (1.7)			
**Secondary measures**									
PANSS positive symptom subscale score									
Placebo	15.4 (0.5)	14.4 (0.5)	14.3 (0.5)	13.9 (0.5)	13.8 (0.5)	12.9 (0.6)	0.3 (0.8)	0.41	.68
BZ	16.0 (0.5)	14.9 (0.5)	13.9 (0.5)	13.3 (0.5)	12.9 (0.5)	12.6 (0.6)			
PANSS negative symptom subscale score									
Placebo	17.6 (0.6)	16.9 (0.6)	16.1 (0.6)	16.3 (0.6)	15.4 (0.6)	15.3 (0.6)	–0.7 (0.9)	–0.79	.43
BZ	17.3 (0.6)	16.9 (0.6)	17.0 (0.6)	17.2 (0.6)	16.4 (0.6)	16.0 (0.6)			
PANSS general psychopathology subscale score									
Placebo	33.9 (0.9)	32.2 (0.9)	30.8 (0.9)	29.4 (1.0)	27.6 (1.0)	27.8 (1.0)	–0.7 (1.4)	–0.49	.63
BZ	34.3 (0.9)	33.1 (0.9)	32.0 (0.9)	30.8 (1.0)	29.4 (1.0)	28.4 (1.0)			
Clinical Global Impression score									
Placebo	3.9 (0.1)	3.8 (0.1)	3.6 (0.1)	3.5 (0.1)	3.4 (0.1)	3.2 (0.1)	–0.4 (0.1)	–2.56	.01
BZ	4.0 (0.1)	4.0 (0.1)	3.9 (0.1)	3.8 (0.1)	3.7 (0.1)	3.6 (0.1)			
Global Assessment of Functioning score									
Placebo	55.6 (1.1)	57.6 (1.1)	59.1 (1.1)	60.2 (1.1)	61.9 (1.1)	63.3 (1.1)	2.1 (1.6)	1.32	.19
BZ	55.4 (1.1)	57.1 (1.1)	57.7 (1.1)	58.8 (1.1)	60.1 (1.1)	61.2 (1.1)			
Hamilton Depression Rating Scale score									
Placebo	7.1 (0.6)	5.4 (0.6)	5.3 (0.7)	4.1 (0.7)	4.5 (0.7)	4.6 (0.7)	–0.8 (1.0)	–0.79	.43
BZ	8.0 (0.6)	8.1 (0.6)	7.9 (0.6)	6.7 (0.7)	6.0 (0.7)	5.3 (0.7)			
Assessment of Quality of Life score									
Placebo	83.6 (1.1)	85.7 (1.2)	87.2 (1.2)	87.5 (1.2)	88.3 (1.2)	88.9 (1.2)	2.4 (1.8)	1.40	.16
BZ	82.2 (1.1)	83.9 (1.2)	83.6 (1.2)	84.9 (1.2)	86.1 (1.2)	86.4 (1.2)			

Abbreviations: BZ, sodium benzoate; PANSS, Positive and Negative Syndrome Scale.

With respect to the secondary clinical measures, there were no significant group differences (*P* < .007) on the 3 PANSS subscales, HDRS, GAF, AQoL, or CGI scales (Table 3). With respect to the per-protocol analyses, there were no significant group differences on the primary nor any of the secondary clinical measures (eTable 3 in Supplement 2).

### Safety

A total of 122 AEs were reported by 66 participants (35 [53%] in BZ group and 31 [47%] in placebo group; individual participants may have experienced >1 AE). Eleven serious AEs were reported by 10 participants, of which only 1 (9%) was assessed as related to the study drug. Again, the rates were comparable between BZ and placebo groups for serious AEs (6 [55%] vs 5 [46%]).

### Amino Acid Concentration in the Peripheral Blood

The concentrations of selected amino acids (and related derived ratios) at baseline and end point are shown in eTable 4 in Supplement 2). We did not find any statistically significant end point change in the concentrations of any of the amino acid–related measures examined in this clinical trial.

## Discussion

There was no significant improvement of total PANSS score in participants with early psychosis after 12 weeks of adjunctive treatment with 1000 mg of BZ per day compared with placebo. Overall, there was no improvements in any of the subscales of PANSS nor in any of the secondary clinical measures. The findings did not change in the planned per-protocol analyses. Our findings provide no support for the hypothesis that BZ is an effective adjunctive treatment for first-episode psychosis.

The lack of efficacy is in contrast to the findings from 2 previous studies.^12,13^ Our protocol differed from these studies on at least 2 key issues. Our sample consisted of people who were relatively young (mean age, 21 years) with recent-onset disorder, while the previous studies were based on participants with longer durations of psychosis and/or treatment resistance. Furthermore, while the previous studies were restricted to participants with schizophrenia, our sample included a broader range of diagnoses; however, most of our participants (83 [84%]) had schizophrenia. Approximately 75% of people with early psychosis achieve symptomatic remission, with affective psychosis having a better prognosis than nonaffective psychosis.^24^ This is consistent with the substantial symptomatic improvements in both the adjunctive BZ and placebo groups in this study.

To explore the putative mechanisms of action of BZ, we examined the peripheral concentration of D-serine, D-alanine (and the proportion of these amino acids of the total D- and L-isomers). We found no difference in these measures by treatment group, which suggests that either BZ had no effect on the metabolism of these amino acids in plasma or the dose used in this study was not sufficient to effect the concentration of amino acids thought to underlie the mechanism of action of BZ. Lin and Colleagues^14^ showed that amino acid plasma levels did not change with BZ even at 2 g/d. While we cannot exclude the possibility that peripheral blood amino acid concentrations may not reflect those found in the central nervous system or that the effect sizes are too small to be detected with this statistical power, our findings cannot reject the hypothesis that higher doses of BZ (ie, those sufficient to alter the amino acid concentration of D-serine and D-alanine) may be clinically useful. There is some evidence to suggest that a higher dose of BZ (eg, 2g/d) is associated with greater improvement in PANSS total score compared with 1g/d.^14^ There is also evidence that the combination of BZ with amino acid derivatives, such as sarcosine, might be beneficial.^12^ Alternatively, BZ may exert effects by other mechanisms. Abnormalities in oxidative stress occur in exacerbations of psychosis,^25^ and agents that modulate oxidative stress, such as N-acetylcysteine, may have value.^26^ Lin and colleagues^14^ found that the symptom improvement was correlated with increases in plasma catalase, which reduces oxidative stress by decomposing hydrogen peroxidase into oxygen and water.

### Limitations

Notwithstanding the strengths of the study (eg, sample size, careful assessment of BZ-related biomarkers), certain limitations may need to be considered. Some other trials of protective agents needed longer times to show efficacy, as long as 6 to 12 months,^27^ and while the trial met its recruitment targets, it was unable to detect small effect sizes. As noted, dose is an area of uncertainty. Furthermore, a large placebo response rate in this trial may have been an issue. Generally, in studies in which there are large placebo response rates, it is unlikely that active agents can separate from placebo. With respect to future research, it would be of interest to undertake a dose-finding study to explore the relationship between daily BZ intake and amino acid concentration in the peripheral blood. The DAAO enzyme is highly expressed in the liver and brain, and it would be expected that BZ would equally effect this enzyme in all tissue compartments.

## Conclusions

This study found no support that adjunctive 1000 mg of BZ per day was of benefit for the treatment of early psychosis. Future clinical trials should restrict participants to those who are treatment refractory and should further investigate whether benzoate acts by altering amino acid levels or by reducing oxidative stress in people with schizophrenia. In the meantime, the routine use of this agent as an adjunctive treatment for early psychosis is not recommended.

## Notes

Supplement 1. **Trial Protocol**

Supplement 2. **eTable 1. **Comparisons of Medications Prescribed in the Benzoate and the Placebo Groups at Baseline **eTable 2. **Comparison of Total Number of Medications Prescribed in the Benzoate and the Placebo Groups at Baseline **eTable 3. **Per-Protocol Analysis: Clinical Measures of All Outcomes Over 12 Weeks Restricted to Those Who Did Not Miss 7 Days of Medication, Average Treatment Adherence of More Than 80%, and Completed the Trial **eTable 4.** End Point Results of Various Amino Acid Levels by Treatment Groups, Adjusting for Baseline Amino Acid Levels

Supplement 3. **Data Sharing Statement**
