# Supplementary material for: Effect of Sodium Benzoate vs Placebo Among Individuals With Early Psychosis: A Randomized Clinical Trial
Source: JAMA Netw Open. 2020 Nov 10;3(11):e2024335. doi: 10.1001/jamanetworkopen.2020.24335 (PMC7656289; doi:10.1001/jamanetworkopen.2020.24335)
Supplement: Supplement 1. — Trial Protocol [file jamanetwopen-e2024335-s001.pdf]

PROTOCOL TITLE

The Efficacy of Sodium Benzoate as an Adjunctive Treatment in Early Psychosis

SHORT TITLE

CADENCE-BZ

Protocol ID: CADENCE-BZ

Version: 1.2
Date: 19th June 2015

SPONSOR

University of Queensland
Brisbane St Lucia, QLD 4072

COORDINATING PRINCIPAL INVESTIGATOR

Professor John McGrath
Queensland Brain Institute
University of Queensland
Brisbane, St Lucia, QLD 4072

AUTHORS

Professor John McGrath- Coordinating Principal Investigator[1]
Associate Professor James Scott – Principal Investigator[1]
Andrea Baker – Study Coordinator[1]

[1]The Park Centre for Mental Health
Wacol QLD 4076 Australia
Phone: 3271 8694

STUDY CENTRE

Queensland Centre for Mental Health Research
The Park Centre for Mental Health
Wacol QLD 4076 Australia
Phone: 3271 8694

STUDY MONITOR

To be advised

DOCUMENT HISTORY

Version	Date	Summary of change
1.0	31st October 2014	Original
1.1	27th April 2015	Additional scales and PICF(participant data register)
1.2	19th June 2015	Staff Training additional PICF, reduction in frequency of scales and correction in dispensation of investigational product.

STUDY ACKNOWLEDGMENT/CONFIDENTIALITY

By signing this Protocol, the Investigator(s) acknowledges and agrees:

The Protocol contains all necessary details for conducting the study. The Investigator will conduct this study as detailed herein, in compliance with Good Clinical Practice and the applicable regulatory requirements, [1] and will make every reasonable effort to complete the study within the time designated.

The Protocol and all relevant information on the drug relating to pre-clinical and prior clinical experience, which was furnished by the Sponsor (the University of Queensland) will be made available to all physicians, nurses and other personnel who participate in the conduct of this study. The Investigator will discuss this material with them to assure that they are fully informed regarding the drug(s) and the conduct of the study.

This document contains information that is privileged or confidential. As such, it may not be disclosed unless specific prior permission is granted in writing by the University of Queensland or such disclosure is required by federal or other laws or regulations. Persons to whom any of this information is to be disclosed must first be informed that the information is confidential. These restrictions on disclosure will apply equally to all future information supplied, which is indicated as privileged or confidential.

The University of Queensland (Sponsor) will have access to source documents entered into the Case Report Form. The Case Report Forms and other data pertinent to this study are the sole property of the University of Queensland (Sponsor), who may utilise the data in various ways, such as for submission to government regulatory authorities, or in publication of the results of the study.

The conduct and results of this study will be kept confidential. The results of this study may be published. Upon completion of the Study, it is the intention of the parties to prepare a joint publication regarding or describing the Study and all the results there from and both parties shall co-operate in this regard.

Coordinating Principal Investigator
Professor John McGrath **Signature:**
Queensland Brain Institute
University of Queensland
Brisbane QLD 4072 Australia **Date:**

Sponsor:
University of Queensland
Signature:

Date:

INVESTIGATORS:

Coordinating Principal Investigator:
Professor John McGrath
Queensland Brain Institute
University of Queensland
Brisbane QLD 4072 Australia
Phone: +61 7 3346 6372
Email: j.mcgrath@uq.edu.au

Principal Investigator:
Associate Professor James Scott
Early Psychosis & Intervention Unit
Level 3 UQ Centre for Clinical Research (UQCCR)
Building 71/918 Royal Brisbane Hospital
Herston QLD 4006
Faculty of Medicine and Biomedical Sciences | University Queensland
Phone: +61 3346 5586
Email: James.Scott@health.qld.gov.au

Associate Investigators
Dr Drew Richardson
The Prince Charles Hospital
Rode Rd
Chermside QLD 4032
Phone: (07) 3139 4000
Email: Drew.Richardson@health.qld.gov.au

Dr Saveena Singh
Gold Coast Hospital
1 Hospital Boulevard
Southport QLD 4215
Phone: (07) 5668 6000
Email: Saveena.Singh@health.qld.gov.au

Dr Frances Dark
Director of Rehabilitation Academic Clinical Unit
Metro South Mental Health Services
519 Kessels Road Macgregor
Upper Mt Gravatt QLD 4122
Phone: 1300 859 998
Email: Frances.Dark@health.qld.gov.au

Dr Sean Hatherill
Clinical Director of Child and Youth Academic Clinical Unit
Logan Hospital
Cnr Armstrong Road & Loganlea Road
Meadowbrook QLD 4131
Phone: (07) 3299 8899
Email: Sean.Hatherill@health.qld.gov.au

Dr Stephen Stathis
Medical Director of Children's Health Queensland CYMHS
Lady Cilento Children's Hospital
Raymond Terrace
South Brisbane QLD 4101
Phone: (07) 3020 8790
Email: Stephen.Stathis@health.qld.gov.au

Dr Sharon Foley
Consultant Psychiatrist
Early Psychosis Team
Metro South Mental Health Services
519 Kessels Road
Macgregor QLD 4109
Phone: (07) 3167 8444
Email: Sharon.Foley@health.qld.gov.au

Table of Contents

1 Introduction 10
1.1 Building clinical trials skills in early psychosis – John Cade Fellowship 10
1.2 Novel interventions for psychosis 10
1.3 Rationale for the Use of Sodium benzoate for the treatment of schizophrenia 11
1.4 Safety profile of sodium benzoate 12
2 Objectives 13
2.1 Primary Objectives 13
2.2 Secondary Objectives 13
3 Study Design 13
4 Study Population 13
4.1 Number of participants 14
4.2 Inclusion Criteria 14
4.3 Exclusion Criteria 14
5 Participant Information and Informed Consent 14
5.1 Screening assessment 15
6 Study Assessments and Procedures 15
6.1 Biomarkers 16
6.2 Study Procedures 19
6.3 Study Restrictions 19
6.4 Safety Assessments 19
6.4.2 Other Safety Assessments 19
6.4.3 Pharmacokinetics 19
6.5 Pharmacodynamics 20
7 Investigational Product 20
7.1 Description of Investigational Product 20
7.2 Dose Justification 21
7.3 Comparator Justification 21
7.4 Administration 21
7.5 Randomisation Procedure 21
7.6 Frequency of visits and follow up 22
7.7 Blinding and Unblinding Procedure 22
7.8 Product Labelling 22
7.9 Handling and Storage of Study Drugs 22
7.10 Accountability 22
8 Adverse Events (AE) and Serious Adverse Events (SAE) 23
8.1 Definition of an Adverse Event (AE) 23
8.2 Definition of a Serious Adverse Event (SAE) 24
8.3 Time Period, Frequency, and Method of Detecting AEs and SAEs 24

8.4 Recording of AEs and SAEs 25
8.5 Prompt Reporting of SAEs 25
8.6 Expeditable Events (SUSAR's) 25
8.7 Evaluating AEs and SAEs 25
8.7.1 Assessment of Intensity 25
8.7.2 Assessment of Causality 26
8.8 Follow-up of AEs and SAEs 26
8.9 Overdose 27
8.9.1 Reporting of Overdose 27
8.10 Pregnancy 27
8.11 Post-study AEs and SAEs 27
8.12 Risk Management Process 28
9 Participant Completion and Withdrawal 35
9.1 Participant Completion 35
9.2 Participant Withdrawal by the Investigator 35
9.3 Participant Withdrawal 35
9.4 Early Termination of the Study 35
10 Case Report Form (CRF) 35
11 Data Analysis and Statistical Considerations 36
11.1 Hypotheses 36
11.2 Endpoints 36
11.2.1 Primary 36
11.2.2 Secondary 36
11.2.3 Tertiary 36
11.3 Sample Size and Power 36
11.4 Statistical Analysis 36
12 Data Management 37
12.1 Documentation 37
12.2 Archiving 37
13 Monitoring and Quality Assurance 37
14 Investigator Responsibility 38
15 Study Report 38
16 Administrative Procedures 39
16.1 Ethical Considerations 39
16.2 Ethical Review Committee 39
16.3 Regulatory Authorities 39
16.4 Informed Consent 39
16.4.1 Adult participants (18-45 years inclusive) 39
16.5 Participant Reimbursement 40

16.6 Emergency Contact with Investigators 40
16.7 Notification of Primary Care Physician and Treating Psychiatrist 40
16.8 Investigator Indemnification 41
16.9 Intellectual Property (IP) and Licencing 41
16.10 Publication Policy 41
16.11 Protocol Amendments 41
16.12 Version Control 42
16.13 Protocol Compliance 42
16.14 Archives: Retention of Study Records 42
17 References 43

ABBREVIATIONS AND DEFINITIONS OF TERMS

AE	Adverse Event
AQOL	Australian Quality of Life
CGI	Clinical Global Impression
CIB	Clinical Investigators' Brochure
CRF	Case Report Form
CTN	Clinical Trial Notification
DAAO	D-amino acid oxidase
GAF	Global Assessment of Functioning
GCP	Good Clinical Practice
HDRS	Hamilton Depression Rating Scale
HHS	Hospital and Health Service
Hr	Hour
HREC	Human Research Ethics Committee
IEC	Independent Ethics Committee
IPCS	International Programme on Chemical Safety
NHMRC	National Health and Medical Research Council
NMDA	N-methyl-D-aspartate
NOAEL	No Observable Adverse Effect Level
PANSS	Positive and Negative Syndrome Scale
PK	Pharmacokinetic
SAE	Serious Adverse Event
SANS	Scale for the Assessment of Negative Symptoms
SD	Standard Deviation
TAU	Treatment as usual
TGA	Therapeutic Goods Administration

1 Introduction

Psychotic disorders are characterized by disturbances to cognition, affect, perception and behaviour. Approximately three percent of the population will be affected by psychosis at some point in their lives; onset is commonly in the second or third decade of life with males at higher risk. While some people will experience a single psychotic episode and recover, many will have persistent symptoms and concomitant cognitive and social disabilities, poor physical health and curtailed life expectancy.[2,3] The pharmacological treatment of psychotic disorders has seen little innovation in recent decades. Current medication focuses primarily on treating psychosis symptoms without addressing concomitant cognitive decline. In addition, current medications used for psychosis are associated with side effects such as weight gain and metabolic syndrome. Pharmacological therapy for psychosis yields inconsistent results with a large proportion of patients only partially responding or remaining treatment resistant.[4] To reduce the personal, social and economic burden associated with psychotic disorders it is of utmost important to investigate novel methods of increasing the efficacy of pharmacological treatment.

1.1 Building clinical trials skills in early psychosis – John Cade Fellowship

Queensland mental health researchers have developed skills in a range of areas (e.g. policy, epidemiology, genetics, imaging), but have been relatively under-represented in the field of clinical trials. In 2013 the NHMRC awarded a John Cade Fellowship to Professor John McGrath in order to build capacity in this field. Headed by Associate Professor James Scott, a team of clinicians, researchers, and mental health consumers are building a clinical trials platform. The platform will focus on finding treatments that benefit people with early psychosis. Candidate treatments will mostly be augmentation studies (i.e. 'add-on' therapies in addition to Treatment as Usual; TAU). The studies will involve masking (e.g. double blind medications) and randomization. There will be shared aspects across the studies (e.g. use of standard outcome measures, minimum data set, and collection of routine biomarkers).

The current study (CADENCE-BZ) is the first of these trials. The title of the trials includes indirect reference to John Cade, the eminent Australian psychiatrist who first proposed that lithium treatments were effective for mania and psychosis. The word "Cadence" has a musical or harmonic connotation that reflects our desire to help patients find better outcomes in their mental health. The BZ suffix stands for sodium benzoate.

1.2 Novel interventions for psychosis

Traditionally pharmacological treatments of psychotic illness predominately focused on modulating the dopaminergic system; however, growing evidence suggests that modulation of glutamatergic system also plays a vital role.[5] N-methyl-D-aspartate (NMDA) receptors can be simplified into two main subunits, the glutamate and glycine binding sites. Options for enhancing NMDA function are limited to agonists or modulators of the glycine binding site, as increasing glutamate levels causes excitotoxicity of neural cells. The D-amino-acids, D-serine and D-alanine, act as a full agonist of the glycine binding site and have shown some promise as an adjunct therapy for treatment of schizophrenia.[6] A recent meta-analysis[6] (2010) found that use of D-serine as an adjunct with antipsychotics was able to improve total psychopathology,(Cohen's d effect size and 95% confidence intervals) 0.40 (0.07-0.73), negative symptoms, 0.48 (0.06-0.90), and notably also improving cognitive symptoms 0.42 (0.12-0.73). D-serine also produced a borderline significant improvement in depressive symptoms 0.39 (-0.01-0.79), however, had no effect on

positive symptoms or general psychopathology. D-alanine has only been investigated in one randomised controlled trial as an adjunct therapy with schizophrenic patients and has shown similar promise to that of D-serine.[7]

A limiting factor with the use of D-serine and D-alanine as an adjunct treatment is they are oxidized by a flavoenzyme, D-amino acid oxidase (DAAO).[8,9] This process both limits the bioavailability of these amino acids, lessening their effectiveness, and increases products that are potentially nephrotoxic in high dosages.[10,11] This limitation has led to interest in compounds that may inhibit the activity of DAAO, and thus lead to an accumulation of endogenous D-alanine. One inhibitor of DAAO is the widely used food preservative sodium benzoate. Experimental animal studies have confirmed that sodium benzoate diminishes DAAO's ability to oxidize D-serine and D-alanine, resulting in increased cerebral concentration of these D-amino acids.[12]

1.3 Rationale for the Use of Sodium benzoate for the treatment of schizophrenia

Taiwanese researchers have recently performed a clinical trial whereby 52 patients with chronic schizophrenia were assigned to adjunct placebo or adjunct sodium benzoate (1000mg per day) treatment for a period of 6 weeks. At the end of the study period all domains of the PANSS had improved by an average of 21% with large effect sizes ranging from 1.16-1.69. The results of the neurocognitive testing were less remarkable with the benzoate group improving their speed of processing (P=.03, ES =0.65) and visual learning and memory (P = 0.02, ES = 0.70), while other domains remained unchanged. Adverse effects were carefully assessed in this trial and no significant differences were noted between the two treatment groups. The benzoate group recorded one case of tachycardia; one case of weight gain; two cases of insomnia. The authors drew the conclusion that these events were likely coincidental observations.[13]

There is also evidence related to the use of sodium benzoate in other mental disorders. A randomized, double-blind, placebo-controlled trial investigating the use of sodium benzoate in patients with Alzheimer disease was identified.[14] 60 patients with amnestic mild cognitive impairment or mild Alzheimer disease were treated with a variable dose of sodium benzoate (250-750mg/day) for a period of 24 weeks. Patients treated with sodium benzoate performed better than placebo on the cognitive subscale of Alzheimer's disease Assessment Scale (p = 0.0031); cognition composite (p =.0007) and clinician interview based impression of change plus caregiver input (p=.012).[14] It was also noted that sodium benzoate was well tolerated without any evident side-effects.

The use of sodium benzoate was also identified in three case studies. Each patient was administered a 6 week trial of sodium benzoate (500mg per day) in the instance where conventional medications were refused by the patient. Two of these cases were drug naïve patients with major depression. Over the 6 weeks course the first patient experienced a reduction of their Hamilton Rating Scale for Depression (Ham-D) score, which fell from 25 to 9, likewise, the second patient with depression had a drop in depression scores after treatment.[15,16] Lastly a patient experiencing panic disorder with somatic symptoms experienced a reduction in the Panic Disorder Severity Scale from 18 to 7 and an associated reduction in somatic symptoms.[17] Although these cases are encouraging it is difficult to differentiate the improvements from a placebo effect.

1.4 Safety profile of sodium benzoate

There is a substantial body of safety information available for sodium benzoate because it has potent antimicrobial properties in acidic conditions; it is an ideal food preservative for products such as salad dressings, carbonated drinks, jams, fruit juices and other condiments. It is also used in some cosmetics and medications. On labels, the inclusion of sodium benzoate is indicated by the code E211. The U.S. FDA has classified sodium benzoate as 'Generally Recognized As Safe' and regulates the concentration of sodium benzoate to 0.1% by weight in food products and 1% concentration in medicines.[18,19] Analysis of orange drinks in England found sodium benzoate at concentrations ranging from 54 to 100mg/litre (mean 76.7mg/litre).[20] Carbonated, water-based, flavoured drinks account for the majority of benzoate intake in Australia/New Zealand, France, the United Kingdom and the USA, whereas, soy sauce was the main contributor of dietary benzoates in China and second largest contributor in Japan.[21] The current acceptable daily intake of 0.5 mg/kg body weight is suggested by the joint committee by the Food and Agriculture Organization of the United Nations and the World Health Organization.[22] It is noted that intake estimations from several counties gave averaged 0.18-2.3mg/kg body weight, however, individuals in China can consume up to 14mg/kg body weight per day from diet alone (i.e. 980 mg per day in a 70 kg person).[21]

The International Programme on Chemical Safety (IPCS) published a report on sodium benzoate (and a related compound benzoic acid) in 2000 detailing the potential health effects of sodium benzoate in animal studies. Testing in rodents revealed a low rate of toxicity with mean lethal dose (LD50) values of >1940mg/kg body weight. Drawing evidence from two long term studies (12-16 months) in rodents, there was no evidence to suggest sodium benzoate had carcinogenic properties. Likewise, studies of the precursors of benzoic acid – benzyl acetate, benzyl alcohol, and benzaldehyde suggest that a carcinogenic effect of sodium benzoate is unlikely. The results of genotoxic activity were inconclusive in the IPCS report, and there was no consistent abnormal findings based on the Ames test. Based on *in vitro* studies of human lymphoblastoid cell lines, the evidence suggests that sodium benzoate at very high concentrations does have genotoxic effects.[23] Sodium benzoate does have embryotoxic and fetotoxic effects, however these are only evident at dosage levels high enough to cause severe maternal toxicity. A No Observable Adverse Effect Level (NOAEL) of approximately 1310 mg/kg body weight for teratogenic effects in rodents was established.[24]

The acute toxicity of oral sodium benzoate in humans is low. There is evidence that some atopic individuals may be sensitive to food additives and preservatives (benzoate is a food preservative).[41] Thus, we will screen and exclude participants with a past history of allergies or intolerance of any food additives.

While the use of sodium benzoate for treatment in psychiatric disorders in humans is a contemporary procedure, it has also been used since the late 1970's to treat patients with urea cycle enzymopathies that cause hyperammonaemia.[25-27] The therapeutic dose administered to treat hyperammonaemia over several years is in the range of 250-500 mg/kg body weight per day, which equates to 17,500-35,000mg per day for a body weight of 70kg. It is noted that at this dose level, the clinical signs of toxicity are rare and are limited to anorexia and vomiting, especially after large intravenous bolus injections with 100% bioavailability.

2 Objectives

Using a randomised, placebo-controlled double-blind parallel-group trial; the primary objective in this study is to examine the clinical efficacy of add-on treatment of sodium benzoate for persistent symptoms in patients with early psychosis.

2.1 Primary Objectives

To determine if 12 week treatment of 1000mg (500mg BD) Sodium Benzoate treatment improves the Positive and Negative Syndrome Scale (PANSS) total score compared to individuals taking placebo.

2.2 Secondary Objectives

To determine if 12 week treatment of 1000mg (500mg BD) Sodium Benzoate treatment improves Positive and Negative Syndrome Scale (PANSS) subscales, Global Assessment of Function (GAF), Australian Quality of Life Scale (AQOL), The Activity and Participation Questionnaire (APQ6), Clinical Global Impression (CGI) and Hamilton Depression rating Scale-17items (HDRS) compared to individuals taking placebo.

2.3 Tertiary (Exploratory) Objectives

To explore if sera markers related to D-alanine, L-alanine, D-serine, L-serine, glycine and glutamate change from baseline to endpoint in those on active treatment.

3 Study Design

The design is a randomised, placebo-controlled, double-blind parallel-group trial to examine the clinical efficacy and safety of add-on treatment of sodium benzoate for persistent symptoms in patients with early psychosis. The study will include 160 individuals with first episode psychosis.

Participants will be given either 1g/d (500mg twice daily) of sodium benzoate or placebo, in addition to their normal routine care. Routine care is defined as 'individualized combinations of psychopharmacology, behavioural interventions, rehabilitation and associated clinical services in keeping with Queensland Health standards of care'

Face to face clinical assessments will be at baseline (week 0) and weeks 2, 4, 6, 8, 10 and 12. Weekly phone contact (if no phone then face to face assessment) will occur in between face to face visits. A post-completion visit will be conducted at week 14. Randomisation will be carried out using a computer-generated randomization table, stratified by five sites. Each of the five sites will have separate randomization tables, and each list will ensure randomization is blocked in groups of four. Participants will receive either active treatment or placebo in a 1:1 ratio.

4 Study Population

One hundred and sixty (160) participants will be recruited through the mental health services in five Queensland Hospital and Health Services: (a) Metro North HHS, (b) Metro South HHS, (c) West Moreton HHS, (d) Gold Coast HHS, and (e) Children's Health Queensland HHS.

4.1 Number of participants

The study will consist of a total of 160 participants.

4.2 Inclusion Criteria

Patients will be invited to participate in the study if they meet all of the following criteria:

1. Aged between 15 and 45 years (inclusive).
2. Fulfil the DSM-IV criteria practice for broadly defined early psychosis, based on the Diagnostic Interview for Psychosis. This includes diagnoses such as schizophrenia, schizophreniform psychosis, delusional disorder, bipolar disorder, psychosis not otherwise specified.
3. Have had the onset of a psychotic disorder within the last two years
4. Have received antipsychotic medications for a period of at least one continuous month within the above two year period.
5. Have a Positive and Negative Syndrome Scale (PANSS) total score of at least 55.
6. Agree to participate, has capacity to consent and able to follow the study instructions and procedures.
7. If under 18 years of age, a parent or legal guardian consents to the young person's participation.

4.3 Exclusion Criteria

Patients will be excluded from the study if they meet any one of the following criteria:

1. Known allergies to sodium benzoate (E211) or any part of the formulation of the investigational product.
2. Suspected allergies or known adverse reactions to food preservatives in general.
3. Comorbid physical illnesses that would impair the participants' ability to complete the trial. (In this 'pragmatic' study, we will not be undertaking additional laboratory testing as inclusion/exclusion criteria. Queensland Health services have routine physical assessment and laboratory testing of patients with Early Psychosis and we will consult with the treating teams about comorbid physical illnesses).
4. People who are unable to understand or communicate in English.
5. For female participant, those currently pregnant, or planning to become pregnant or lactating during the study period.
6. Inability to follow the study instructions and procedures.

5 Participant Information and Informed Consent

Consent will only be obtained from patients who are deemed to have capacity to provide informed consent. Capacity will be determined by collaboration between the treating clinician and delegated research assistant and will comply with the guidelines within the NHMRC National Statement on Ethical Conduct in Human Research 2007. Participants under 18 years of age require parent or legal guardian consent to participate.

Under 4.5.8 of the National Statement, people with a mental illness, "consent should be witnessed by a person who has the capacity to understand the merits, risks and procedures of the research, is independent of the research team and, where possible, knows the participant and is familiar with his or her condition" (e.g. Treating Clinician). We will ensure that a witness also signs the consent form. In the event where the research assistant is unable to find a witness who is familiar with the patient, an independent witness will be used for this process.

During the consenting process, all participants (including parent or legal guardian) will be informed that they have the right to withdraw consent from the study at any time without prejudice and withdrawal from the study will not affect their current or future care. Revocation of consent forms will be completed for those participants who choose to withdraw from the study.

5.1 Screening assessment

After verbal consent is provided, an assessment of inclusion/exclusion criteria will commence. Participants who meet all inclusion criteria and none of the exclusion criteria will be invited to participate in the study and the formal consent process will commence. For those who consent to participate, they will be enrolled in the study and randomized.

It is important to acknowledge participants' enthusiasm and interest in research. Participants who do not meet the inclusion criteria will therefore, be invited to be registered on the Queensland Centre for Mental Health Research (QCMHR) participant data register. A separate patient information consent form will be provided to those participants who are interested in this option.

6 Study Assessments and Procedures

To maintain valid interrater reliability for this study we will be initially conducting a training phase to ensure research staff are competent in the accurate administration of the Positive and Negative Syndrome Scale (PANSS) and the Diagnostic Interview for Psychosis. The study population will be recruited using the same methods as per section 4 (Study Population) of this protocol. The training phase will consist of 30 participants aged between 18-45 years with a diagnosis of schizophrenia. Informed consent will be obtained as per section 5 (Participant Information and Informed Consent) of this protocol.

For the main study a battery of validated clinical measures, physical health measures (blood pressure, waist circumference, height, weight and Body Mass Index (BMI)) and adverse events will be conducted at baseline, weeks, 2, 4, 6, 8, 10 and 12.

Efficacy measures include:
Positive and Negative Syndrome Scale (PANSS) total score will be used as the primary outcome measure which is a widely used scale for measuring symptom severity of patients with schizophrenia.

Secondary outcome measures will include the following clinical assessments:

- PANSS subscales including Positive, Negative and General Psychopathology.

- Global Assessment of Function (GAF) which is a numeric scale (1 through 100) used by mental health clinicians and physicians to rate subjectively the social, occupational, and psychological functioning of adults.

- Clinical Global Impression (CGI) which is used to measure symptom severity, treatment response and the efficacy of treatments in treatment studies of patients with schizophrenia.

- Hamilton Depression rating Scale-17items (HDRS) is a multiple item questionnaire used to provide an indication of depression, and as a guide to evaluate recovery.

- Australian Quality of Life Scale (AQOL) is a 15 item instrument that measures five broad domains: Psychological well-being, physical senses, social relationships, independent living, and illness.

- Patient Global Impression (PGI) is a global index that is used to rate the response of a condition to a therapy (transition scale).

- Opiate Treatment Index (OTI) is a structured interview designed to provide a measure of the effectiveness of drug treatments.

- International Physical Activity Questionnaires (IPAQ) provides a common instrument that can be used to obtain internationally comparable data on health–related physical activity.

- The “Simple Physical Activity Questionnaire” (SIMPAQ) measures physical activity. It has been designed for use in various populations including clinical samples with high levels of sedentary behaviour.

- The Physical Activity Questionnaire (PAQ) has been developed to assess attitudes. Specifically, assessing; general opinion on physical exercise, motivators and barriers to exercise.

- The Activity and Participation Questionnaire (APQ6) is a short 6 item measure designed to complement clinical assessment, to support dialogue between consumers and clinicians about recovery goals, and to allow monitoring of change over time at both individual and aggregated service levels.[28]

6.1 Biomarkers

The Director of the National Institute of Mental Health (Dr Tom Insel) has recently urged that clinical trials in mental health need to be enriched for biomarkers related to the proposed “target” of the intervention. This will be achieved by collecting a 25ml blood sample via venipuncture at both baseline and final patient interviews. A portion of this sample will be used to assess the concentration of key analytes in the pathways related to sodium benzoate. D-amino acid oxidase is needed to catabolize (break down) D-alanine. D-alanine can be measured in sera with High Performance Liquid Chromatography HPLC.[29] In addition; we can measure related species that could be altered by the intervention, including L-alanine, D- and L-serine, glycine and glutamate. The investigators have suitable equipment and laboratory skills to assess these molecules. Collecting these measures will allow us to assess if sodium benzoate impacts on these analytes in the peripheral blood.

The remainder of the blood sample will be stored indefinitely for future research. However, future studies involving the stored blood samples will require approval from a Human Research Ethics Committee. There is strong justification to explore the association between common variants in genes involved in the glutaminergic pathways (benzoate impacts on analytes that are involved in these systems). For example, the recently published study in Nature identified 108 loci linked to

risk of schizophrenia.[30] Several of these are linked to genes in these same pathways that are related to the action of sodium benzoate Of particular relevance to the current protocol, common variants in serine racemase (SRR were significantly associated with an increased risk of schizophrenia – this enzyme is upstream of DAAO, and involved in the metabolism of serine. While the study of the current sample size will not have sufficient power to test the impact of common variants in these genes versus outcomes in this study (the effect sizes for all SNPs linked to schizophrenia are small, between 1.1 and 1.4), we wish to bank this material down for future collaborative studies. This feature of the study will be clearly outlined in the consent form, and participants will be informed that DNA and sera samples may be used in future years by national and international collaborative, after approval from a Human Research Ethics Committee.

In the event that a person objects to any biological samples being collected and stored, they will not be excluded from participating in the trial.

Table 1: Schedule of Visits and Assessments

Assessment schedule can vary plus or minus five days for operational convenience

**Visit**	0 Screening Phase	1 Baseline	2	3	4	5	6	7	8	9	10	11	12	13	14
**WEEK**		0	1	2	3	4	5	6	7	8	9	10	11	12	14
**Study medication period(12 weeks)**															
**SCREENING AND CONSENT**															
Assessment of current medication	X														
Informed consent	X														
Ongoing capacity		X	X	X	X	X	X	X	X	X	X	X	X	X	
Inclusion / exclusion criteria	X														
Urine pregnancy test (females only)	X														
Drug dispensation (after randomisation)		X		X		X		X		X		X			
**SAFETY**															
Adverse events				X	X	X	X	X	X	X	X	X	X	X	X
Physical health and metabolic measures		X		X		X		X		X		X		X	
**EFFICACY**															
PANSS TOTAL SCORE	X	X		X		X		X		X		X		X	
GAF		X		X		X		X		X		X		X	
AQOL		X		X		X		X		X		X		X	
APQ-6		X												X	
CGI		X		X		X		X		X		X		X	
HDRS		X		X		X		X		X		X		X	
PGI														X	
OTI		X				X				X				X	
IPAQ		X													
SIMPAQ			X												
PAQ				X											
**OTHER**															
Drug compliance			X	X	X	X	X	X	X	X	X	X	X	X	
Blood test (DNA extraction, , sera storage)		X												X	

6.2 Study Procedures

Dispensing of sodium benzoate to participants will occur once consent has been obtained and after the screening phase and randomisation has occurred. A delegated Research Pharmacist at the Princess Alexandra Hospital will dispense medication for all sites. For each randomised participant, the entire 12 weeks of study medication will be provided to QCMHR delegated research staff. The study medication will then be distributed to the participant on a fortnightly basis by delegated research staff in line with this protocol (section 7.4). There will be a total of 6 dispensations per participant.

6.3 Study Restrictions

There are no restrictions[43] to participants during the study in terms of concomitant medications, exercise or ambulation. While sodium benzoate is often added to vitamin C rich products such as orange juice, there has been concern that sodium benzoate may interact with high dose vitamin C formulations to produce benzene (a known carcinogen). There is an absence of clear guidance on this matter, thus for the purposes of this protocol, we will adopt a conservative stance and require patients to avoid using vitamin C formulations that contain more than 500 mg/day (multivitamin tablets have lower doses of vitamin C and can be continued during the study, as can citrus fruits and food products that contain vitamin C naturally).

6.4 Safety Assessments

All patients recruited in this study will be active cases at Queensland Hospital and Health services. The study team will liaise with clinical staff to ensure that participants have undergone a routine physical health screen.

Female participants will have a urinary pregnancy screen at baseline prior to inclusion, and during the study if indicated.

6.4.1 Adverse Events

The Investigator and designated study personnel will monitor each participant for adverse events during the study. All adverse events reported between consent and final follow-up visit will be recorded in the case report form (CRF). The investigator or designee will ask the participant non-leading questions in an effort to detect adverse events e.g. “Have you felt unwell or different in any way since your last visit”.

In addition, participants will be encouraged to spontaneously report any unusual feelings or sensations. See Section 8 for full details on adverse event reporting.

6.4.2 Other Safety Assessments

If the participant is of child-bearing potential and sexually active, urine pregnancy tests will be conducted at baseline or when clinically appropriate.

6.4.3 Pharmacokinetics

The following information is sourced directly from the Concise International Chemical Assessment Document 26 “Benzoic Acid and Sodium Benzoate”, World Health Organization.

After oral ingestion of benzoic acid and sodium benzoate, there is a rapid absorption (of undissociated benzoic acid) from the gastrointestinal tract in experimental animals or humans.[31,32] One hundred percent absorption can be assumed from the general literature when sodium benzoate is administered orally. In humans, the peak plasma concentration is reached within 1–2 hours [33,34]. In the acid conditions of the stomach, the equilibrium moves to the undissociated benzoic acid molecule, which is believed to be absorbed rapidly. Benzoate from sodium benzoate changes from the ionized form to the undissociated benzoic acid molecule. As a result, the metabolism and systemic/toxic effects of benzoic acid and sodium benzoate can be evaluated together.

After oral and dermal uptake, sodium benzoate is metabolized in the liver by conjugation with glycine, resulting in the formation of hippuric acid.[22,31,35,36] The rate of biotransformation in humans is high: after oral doses of 40, 80 or 160 mg sodium benzoate/kg body weight, the transformation to hippuric acid was independent of the dose — about 17–29 mg/kg body weight per hour, corresponding to about 500 mg/kg body weight per day.[34] Other authors obtained higher values of 0.8–2 g/kg body weight per day. [31,32] Hippuric acid is rapidly excreted in urine. In humans, after oral doses of up to 160 mg/kg body weight, 75–100% of the applied dose is excreted as hippuric acid within 6 hours after administration, and the rest within 2–3 days.[33,34,37]

The limiting factor in the biosynthesis of hippuric acid is the availability of glycine. The utilization of glycine in the detoxification of benzoate results in a reduction in the glycine level of the body. Therefore, the ingestion of benzoic acid or its salts affects any body function or metabolic process in which glycine is involved; for example, it leads to a reduction in creatinine, glutamine, urea, and uric acid levels. [22,31,32,34]

Another metabolite of benzoate is the benzoyl glucuronide. For example, the dog excretes considerable amounts of this metabolite in the urine (20% after a single dose of 50 mg/kg body weight).[38] In healthy adults, up to 97% of the dose of sodium benzoate is excreted as hippuric acid in the urine within 6 hours

6.5 Pharmacodynamics

The desired pharmocodynamic outcome of sodium benzoate relates to its ability to inhibit D-amino-acid-oxidase (DAAO). DAAO is an enzyme that metabolizes D-amino-acids, such as D-serine and D-alanine. Sodium benzoate binds to the D-amino-acid-oxidase active site, which inhibits its ability to metabolize D-amino-acids. Consequently, by inhibiting DAAO, this results in increased concentrations D-amino acids. As D-serine and D-alanine are full agonists of the glycine binding site of the NMDA receptor, the increased cerebral concentrations of these D-amino acids result in increased NMDA function.

7 Investigational Product

7.1 Description of Investigational Product

Active: Sodium benzoate 500 mg per gelatine capsule administered at 1000mg per day (500mg BD)
Placebo: Microcrystalline cellulose in matched gelatine capsule

7.2 Dose Justification

With respect to the use of sodium benzoate as a food, the Joint Food and Agriculture Organization of the United Nations (FAO) / World Health Organization (WHO) Expert Committee on Food Additives, established a preliminary acceptable daily intake of up to 5mg/kg body weight for sodium benzoate.[22] This translates to 350mg per day for a 70kg person.

With respect to individuals with urea cycle disorders, the recommended dose of sodium benzoate is between 250-500mg/kg per day or approximately 17500mg-35000mg per day for a 70kg person. It is noted that serious side effects at this dosage are rare and limited to occasional cases of anorexia and vomiting.[39]

With respect to the use of sodium benzoate for the treatment of schizophrenia, we are guided by the dose used on Lane and colleagues (2013). In the main study the investigators also described a pilot study (n=7) dosing sodium benzoate between 250 and 1000mg/d. 1000 mg/d resulted in the largest decrease in psychotic symptomatology with no evident adverse reactions. In this patient group, a dose of 1000 mg per day was not associated with significantly more adverse events compared to placebo. Treatment-emergent adverse events included weight gain (n=1), insomnia (n=2), and tachycardia (n=1). It was noted that these adverse events were mild, brief and did not warrant medical treatment. The authors concluded these events were likely coincidental observations. Routine blood cell count, chemical analysis results and electrocardiogram after treatment remained unchanged and were all within normal ranges.[13]

7.3 Comparator Justification

This study will use a placebo adjunct to routine care (routine care in this study is defined as 'individualized combinations of psychopharmacology, behavioural interventions, rehabilitation and associated clinical services in keeping with Queensland Health standards of care for psychosis') as a comparator condition. The Declaration of Helsinki affirms that placebo-controlled trials should only be used in the absence of existing proven therapy.[40] Therefore the use of an adjunct therapy has been selected to ameliorate these ethical concerns as both the experimental and control groups will receive standard medical care (Treatment as Usual).

7.4 Administration

Sodium Benzoate – 1000mg (500mg BD with meals-reminder aid) capsules will be used in the study.
Placebo – identical-appearing microcrystalline cellulose gelatine capsules will be used in the study.

7.5 Randomisation Procedure

Participants will be randomised once written consent has been obtained and the baseline assessments have been completed. Participants will be randomised to one of the treatment groups, using blocks of 4 via a computer-generated randomization table. Randomisation will be stratified by five sites (a) Metro North HHS, (b) Metro South HHS, (c) West Moreton HHS, (d) Gold Coast HHS, and (e) Children's Health Queensland HHS. Participants will receive either active treatment or placebo in a 1:1 ratio.

The investigational products will be manufactured in accordance with current Good Manufacturing Practice (GMP) in a suitable TGA licensed facility. This same company (Pharmaceutical Packing Professionals) will hold the randomisation code, and provide a 24 hour

1800 number to unblind participants if required. Participants will be randomised strictly using a chronological process. Participants will be allocated a unique identification number which will be linked to the specific site number. If a participant withdraws from the study then the participant number will not be re-used nor will the participant be allowed to re-enter the study.

The randomisation will be double-blind. An independent Biostatistician will generate the randomisation list which will be provided to the manufacturer. The independent manufacturer will hold the closed randomisation list and be the only one who has the ability to unblind. In the case of emergency where it is crucial the medical staff knows whether the participant is on sodium benzoate or placebo, participants will be provided with contact information (i.e. 24 hour 1800 number) for unblinding.

7.6 Frequency of visits and follow up

Participants will be clinically assessed at baseline, week 2, 4, 6, 8, 10 and 12. The study team will also contact participants once a week between face-to-face assessments by phone (if no phone then face to face assessment). Refer to Table 1 Schedule of Visits and Assessments.

7.7 Blinding and Unblinding Procedure

All medication will be blinded to the study personnel, research pharmacist and the patient. Sodium benzoate and placebo capsules will be identical in packaging, appearance, colour and taste. Treatment allocations will not be disclosed to the Investigator or any study personnel before the database is locked, unless in the case of an emergency requiring unblinding. Unblinded participants will be withdrawn from the study.

Only in the event of a medical emergency which the investigator feels cannot be adequately managed without knowing the identity of the study medication, will the treatment code be unblinded for a particular participant. This will be done by the independent manufacturer via the 24 hour 1800 number. All cases of emergency unblinding will be documented on a Serious AE Form and reported to The University of Queensland, (Sponsor) within 24 hours.

After the completion of all participants in the study (last patient last visit), participants will be notified which arm of the study they took part in.

7.8 Product Labelling

The labelling of study medication will comply with local regulatory GCP and TGA requirements and medication dispensing guidelines.

7.9 Handling and Storage of Study Drugs

Prior to dispensing, all study medication will be kept securely locked, in a dry, restricted access location at room temperature (20-25°C). Only delegated members of the study team will have access to the investigational products.

7.10 Accountability

The designated Research Pharmacist will dispense study medication into the care of the delegated research staff, who will then sign that he/she has received the study medication for the study. The study drug will be kept in a securely locked area and provided to the participants according to the protocol (section 7.4). Participants will be requested to return all unused study medication (i.e.

unopened blister packs or capsules not taken) and empty blister packs to the delegated research assistants. All unused supplies of study medication will be accounted for and documented by the designated Research Pharmacist. Compliance with study medication will be calculated at each visit by means of self-report and a capsule count. This data will be used to calculate compliance with medication for analysis purposes. To further aid compliance and as a quick visual guide, participants will be offered a diary to document the date and time of scheduled visits and tasks to be undertaken.

All material supplied is for use only in this clinical study and should not be used for any other purpose. The Investigator is responsible for investigational product accountability, reconciliation and record maintenance. In accordance with all applicable regulatory requirements, the Investigator or designated site staff will maintain investigational product accountability records throughout the course of the study. These persons will document the amount of investigational product received from the Sponsor, the amount supplied and/or administered to and returned by participants, if applicable.

An investigational product dispensing Log will be kept current and will contain the following information:

- the identification of the participant to whom the drug was dispensed;
- the date(s) and quantity of the drug dispensed to the participant.

The inventory will be available for inspection by study monitors during the study. Drug supplies including participant returns will be collected at the end of the study by the study monitor, returned by the Investigator or designee to the Sponsor or authorised for destruction. When requested in writing by the Sponsor, unused drug supplies may be destroyed by the Investigator or delegate provided such disposition does not expose humans to risks from the drug. Records will be maintained by the Investigator of any such alternate disposition of the investigational product. These records will show the identification and quantity of each unit disposed of, the method of destruction (taking into account the requirements of local law), and the person who disposed of the investigational product. Where investigational product is destroyed on-site, a record of destruction will be issued. Such records will be submitted to the Sponsor for reconciliation purposes.

8 Adverse Events (AE) and Serious Adverse Events (SAE)

The investigator will be responsible for the detection and documentation of events meeting the criteria and definition of an adverse event (AE) or a serious adverse event (SAE) as provided in section 8.1. During the study, when there is a safety evaluation, the investigator or delegated research staff will be responsible for detecting AEs and SAEs, as detailed in this section of the protocol.

8.1 Definition of an Adverse Event (AE)

Any untoward medical occurrence in a participant or clinical investigation participant, temporarily associated with the use of a medicinal product, whether or not considered related to the medicinal product.

For the current study, an AE is defined as any unfavourable and unintended sign (including an abnormal laboratory finding), symptom, or disease (new or exacerbated) temporally associated with the use of a medicinal product, whether or not considered related to the medicinal product.

Examples of an AE **include**:

- Exacerbation of a chronic or intermittent pre-existing condition including either an increase in frequency and/or intensity of the condition.
- New conditions detected or diagnosed after investigational product administration even though it may have been present prior to the start of the study.
- Signs, symptoms, or the clinical sequelae of a suspected interaction.
- Signs, symptoms, or the clinical sequelae of a suspected overdose of either investigational product or a concurrent medication (overdose *per se* should not be reported as an AE/SAE).
- Acute episode of psychosis

Examples of an AE **do not include** a/an:

- Medical or surgical procedure (e.g. endoscopy, appendectomy); the condition that <u>leads</u> to the procedure is an AE.
- Situations where an untoward medical occurrence did not occur (social and/or convenience admission to hospital).

In this study, AEs may include the following documented side effects: Anorexia, vomiting, allergic reactions

8.2 Definition of a Serious Adverse Event (SAE)

A serious adverse event is any untoward medical occurrence that, at any dose:
a) results in death
b) is life threatening
c) requires hospitalisation or prolongation of an existing hospitalisation.
Hospitalisation for elective treatment of a pre-existing condition that did not worsen from baseline is not considered an AE.
d) results in disability/incapacity, or
e) is a congenital abnormality / birth defect.
f) Any event deemed by the investigator as being a significant medical event.

8.3 Time Period, Frequency, and Method of Detecting AEs and SAEs

All adverse events will be recorded between the time of consent and the final visit (week 14), two weeks post final treatment. Each Participant will be monitored regularly by the investigator and study personnel for adverse events occurring throughout the study. The research assistant will enquire about AEs by asking the following non-leading questions:

At the first scheduled visit (pre-dosing) participants will be asked:
"How are you feeling?"
At subsequent scheduled visits, participants will be asked:
"Since your last visit, have you had any health problems?"

8.4 Recording of AEs and SAEs

When an AE/SAE occurs, the investigator or delegate will review all documentation (e.g. hospital progress notes, laboratory, and diagnostic reports) relative to the event. The investigator or delegate will then record all relevant information regarding an AE/SAE in to the CRF.

For each adverse event, start and stop dates, action taken, outcome, intensity (see Section 8.7.1) and relationship to study product (causality) (see Section 8.7.2) will be documented. If an AE changes in frequency or intensity during a study, a new entry of the event will be made in the CRF. All details of any treatments initiated including concomitant medications due to the adverse event will be recorded in the Case Report Form (CRF).

8.5 Prompt Reporting of SAEs

Once an investigator becomes aware that an SAE has occurred in a study Participant, he/she will immediately notify the University of Queensland (sponsor) by contacting the study monitor via telephone to notify him/her of the event. The SAE form must be completed as thoroughly as possible with all available details of the event, signed by the investigator (or appropriately qualified designee), and faxed to the study monitor within 24 hours of first becoming aware of the event.

If the investigator does not have all information regarding an SAE, ***he/she will not wait to receive additional information before notifying the study monitor*** of the event and completing the form. The form will be updated when additional information is received.

The investigator will always provide an assessment of causality at the time of the initial report as described in Section 8.7.2, “Assessment of Causality”. If data obtained after reporting indicates that the assessment of causality is incorrect, then the SAE form may be appropriately amended, signed and dated, and resubmitted to the Sponsor.

In accordance with current QH guidelines, the investigator must also notify the Reviewing Ethics Committee or site governance Office of any SAEs according to the guidelines of the Ethics Committee.

8.6 Expeditable Events (SUSAR’s)

Expeditable events are those adverse events that are **CAUSALLY** related to the study product, **AND** that are both **SERIOUS** (see Section 8.2) and **UNEXPECTED** (see Section 8.7.3). These events are deemed Suspected Unexpected Serious Adverse Reactions. Reporting timeframes to the TGA and other regulators will be conducted in accordance with the relevant guidelines.

8.7 Evaluating AEs and SAEs

8.7.1 Assessment of Intensity

The investigator will make an assessment of intensity for each AE and SAE reported during the study. The assessment will be based on the investigator’s clinical judgement. The intensity of each AE and SAE recorded in the Case Report Form (CRF) will be assigned to one of the following categories:

Mild: An event that is easily tolerated by the Participant, causing minimal discomfort and not interfering with everyday activities.
Moderate: An event that is sufficiently discomforting to interfere with normal everyday activities.
Severe: An event which is incapacitating and prevents normal everyday activities.

An AE that is assessed as severe should not be confused with an SAE. Severity is a category utilised for rating the intensity of an event; and both AEs and SAEs can be assessed as severe. An event is defined as "serious" when it meets one of the pre-defined outcomes as described in Section 8.2 "Definition of an SAE".

8.7.2 Assessment of Causality

The investigator will assess the relationship between investigational product and the occurrence of each AE/SAE. The investigator will use clinical judgment to determine the relationship. Alternative causes, such as natural history of the underlying diseases, concomitant therapy, other risk factors, and the temporal relationship of the event to the investigational product will be considered and investigated.

The causal relationship to the study product assessed by the Investigator (or medically qualified delegate) will be assessed using the following classifications:

Not Related In the Investigator's opinion, there is not a causal relationship between the study product and the adverse event.
Unlikely The temporal association between the adverse event and study product is such that the study product is not likely to have any reasonable association with the adverse event.
Possible The adverse event could have been caused by the study Participant's clinical state or the study product.
Probable The adverse event follows a reasonable temporal sequence from the time of study product administration, abates upon discontinuation of the study product and cannot be reasonably explained by the known characteristics of the study Participant's clinical state.
Definitely The adverse event follows a reasonable temporal sequence from the time of study product administration or reappears when study product is reintroduced.

8.7.3 Assessment of Expectedness

Expected An adverse reaction, the nature or severity of which is consistent with the applicable product information (e.g. Investigators' Brochure) for an unapproved medicinal product).
Unexpected An adverse reaction, the nature or severity of which is not consistent with information in the relevant document (e.g. Investigators' Brochure for an unapproved medicinal product).

8.8 Follow-up of AEs and SAEs

All AEs and SAEs documented at a previous visit/contact and are designated as ongoing, will be reviewed at subsequent visits/contacts.

All AEs and SAEs will be followed until resolution, until the condition stabilises, until the event is otherwise explained, or until the Participant is lost to follow-up. Once resolved, the appropriate AE/SAE Case report Form (CRF) page(s) will be updated.

8.9 Overdose

An overdose is defined as a dose taken by a patient in excess of the doses in the approved study protocol or available product information, either accidentally or intentionally, irrespective of whether it involves study medication or non-study medication. Overdose may be suspected or confirmed and may or may not be associated with clinical signs and symptoms.

It would definitely include (but not be limited to) those events which based on the investigators clinical judgment were considered to be of medical concern and /or require clinical observation and /or medical intervention. An overdose would include any dose greater than the highest daily dose included in the protocol or available product information. Deviations to study drug administration (i.e. resulting from poor patient compliance) which do not meet the definition of an overdose, will be recorded in the study medication compliance section of the case Report Form (CRF) and not as Serious AE's.

8.9.1 Reporting of Overdose

For all overdoses the Serious AE Form will be completed and reported to the sponsor within 24 hours from the time that the Investigator or delegated research staff have been made aware of the event. See section 8.5 for all other Serious AEs. The documentation will include details of any associated signs/symptoms or if the overdose is asymptomatic, this will be stated.

8.10 Pregnancy

Details of all pregnancies in participants that occur during the treatment period and the final follow-up visit will be documented and reported to the Investigator. In addition, any pregnancies brought to the attention of the Investigator after this period, and where it is known that study medication was taken at the time of conception, will also be reported.

Although pregnancies are not generally serious AE's, the Serious AE Form will be completed and forwarded to the Investigator within 24 hours. This will provide a record of the initial notification of the pregnancy.

Pregnancy is an exclusion criterion for this study, therefore, participants who become pregnant during the study should discontinue the study medication immediately and will be withdrawn from the study. The Investigator or delegated research staff will contact the participants treating Physician and inform them of the pregnancy in writing.

8.11 Post-study AEs and SAEs

A post-study AE/SAE is defined as any event that occurs outside the AE/SAE detection period as defined in Section 8.3 "Time Period, Frequency, and Method of Detecting AEs and SAEs" of the protocol.

For participants that have experienced AE's and SAE's during the trial, we will follow-up until resolution and/or liaise with the treating team to optimize ongoing care as appropriate.

8.12 Risk Management Process

Table 2 below details the Risk Identification, Evaluation and Management plan for this study. It will ensure that risk and uncertainly are appropriately managed for the duration of the study. The risk management process is in accordance with the NHMRC National Statement on Ethical Conduct in Research Involving Humans (2007).

Table 2: Risk Analysis Matrix

Consequence

**Likelihood**	**Negligible**	**Minor**	**Moderate**	**Major**	**Extreme**
Almost Certain					
Likely					
Possible					
Unlikely					
Rare					

Response To Risk

	Very High	Immediate action required
	High	Urgent attention or investigation required
	Medium	Require specific attention
	Low	Manage through routine procedures

Risk Identification, Evaluation and Management Plan

	Risk	Description	Possible Effects			Risk Management strategies
			Likelihood	Consequence	Rating	
1.	Psychological discomfort during interview	Participants may experience psychological discomfort when answering questions in the clinical interview	Possible	Minor-moderate	Medium	The PICF clearly states the potential risk of discomfort.<br><br>Recruitment of experienced mental health clinicians who will be able to minimise and manage discomfort.<br><br>Participants will be clinically assessed at baseline, and every second week for 12 weeks. We will also contact participants once a week between face-to-face assessments by phone or other electronic means where participants are given the

						opportunity to discuss any concerns/discomforts re previous appointment.<br><br>Clinicians will direct and assist participants to gain support if required.
2.	Inconvenience of participating in the trial	Participants may be inconvenienced by time taken to participate in the trial.	Possible	Negligible	Low	The PICF clearly states the battery of clinical assessments to be completed and the approximate time and frequency for clinical assessment visits.<br><br>Participants will be given as many breaks as necessary throughout the clinical assessment visit.<br><br>Participants will be reimbursed for their time involved in the trial.<br><br>Participants will be reminded that the trial is voluntary and they can withdraw at any time.
3.	History of self-harm/suicidal ideation	Participant expresses suicidal ideation.	Possible	Moderate-severe	High	Recruitment of experienced mental health clinicians who are trained in conducting risk assessment and managing high risk situations.<br><br>Research staff will have access to

						a clinically trained senior staff including a Project Manager and Chief Investigator who will assist research staff to conduct risk assessment and implement risk management plan if required i.e. notifying treating team and assisting in the participant accessing appropriate support (e.g. emergency services)<br><br>Previously identified high risk patients and recent risk assessments will be discussed at weekly team meetings and their management reviewed by senior research staff (including Project Manager and Chief Investigator).<br><br>Research staff will be given support and feedback on risk assessments and their management to improve skills throughout the project.
4.	Blood test	Two blood samples will be taken from participants who consent to the procedure. Participants may experience some short term mild discomfort from the blood draw. Participants may	Possible	Negligible	Low	The PICF clearly states the potential complications associated with the blood draws. Participants provide specific consent for this procedure which is identified on the consent form.

		experience minor complications such as local bruising and inflammation of the vein used.				Participants will be taken to recognised pathologies who have trained phlebotomists to conduct the blood draws.<br><br>Participants are made aware they can refuse a blood draw at any stage throughout the study.
5.	Overdose	An overdose would include any dose greater than the highest daily dose included in the protocol or prescribing information.	Possible	Minor-Moderate	Medium-High	For all overdoses the Serious AE Form will be completed and reported to the sponsor within 24 hours from the time that the Investigator or delegated research staff was notified of the overdose.<br><br>Participants will be provided with 14 days' supply (blister pack) at each face to face visit.<br><br>Research staff will conduct a pill count at each face to face visit.<br><br>Research staff will conduct medication compliance questionnaire at each face to face visit and phone contact.<br><br>Any identified issues with medication compliance will be discussed at weekly team meetings. Senior research staff

						(including Project Manager and Chief Investigator) will determine the most appropriate plan of action if required.
6.	Home visits	Participants may be seen at home rather than in the clinic.<br>Individuals with psychosis can often experience hallucinations and delusions which could result in unpredictable behaviour.	Possible	Minor-Moderate	Medium-High	First preference should be interview conducted at the clinic in a suitable interview room.<br><br>Two staff will be required for home visits and will carry a mobile phone.<br><br>Research staff will adhere to a sign in/out policy and advise the Project Manager of the address they will be attending.<br><br>Any incidents from a home visit will be reported to the Project Manager and Chief Investigator and documented in the CRF or if required reported to Metro South HREC.
7.	Transporting participants in QLD Health work vehicles	Research staff will be transporting participants to pathology appointments and may be required to transport participants to the interview site.<br>- There may be risk associated with	Possible	Minor-Moderate	Medium -High	Research staff will have a current QLD Driver's Licence and completed the mandatory Driver Safety E-Learning Course.<br><br>Recruitment of experienced mental health clinicians who will be able to and manage

		motor vehicle accident<br>- There may be risks associated with unpredictable behaviour of a patient whilst being transported.				unpredictable behaviour.<br><br>Research staff will carry a mobile phone and adhere to a sign in/out policy and advise the Project Manager of the address they will be attending.

9 Participant Completion and Withdrawal

9.1 Participant Completion

Participants are considered to have completed the study if they complete 12 weeks of dosing.

9.2 Participant Withdrawal by the Investigator

Patients will be withdrawn from the study by the Investigator, prior to completion of treatment, under the following conditions:

- Non-compliant with study medication for seven consecutive days
- Development of a serious adverse event assumed to be associated with the study medication
- Cessation of effective contraception or confirmed pregnancy
- Continual inability to provide informed consent.

9.3 Participant Withdrawal

All participants have the right to withdraw consent at any time without prejudice and this will not affect their ongoing care. This will be clearly discussed during the consenting process. If a participant decides to withdraw consent we will complete a revocation of informed consent form.

9.4 Early Termination of the Study

The study may be terminated prematurely by the Coordinating and or Principal investigator or his/her designee and the sponsor if:

- The number and/or severity of adverse events justify discontinuation of the study.
- New data becomes available which raises concern about the safety of the study drug, so that continuation might cause unacceptable risks to participants.

After such a decision, the Investigator or designee will contact all participants promptly, and written notification of study termination will be sent to the Reviewing Ethics Committee and relevant Governance Offices. A study closure advice will also be sent to the TGA on the approved form. The Australian Clinical Trial Registry entry will also be updated accordingly.

10 Case Report Form (CRF)

A Case Report Form (CRF) will be completed for each study participant summarising all clinical screening and study data that is to be provided to the University of Queensland (Sponsor) for data analysis. In the CRF, participants will only be identified by their participant number in order to retain participant confidentiality.

The completed Case report Forms (CRF's) will be retained by the Investigators for a period of at least 15 years or the maximum time frame as determined by local regulations, whichever is the longest.

11 Data Analysis and Statistical Considerations

11.1 Hypotheses

Those participants allocated to the active arm (1000mg (500mg BD)) Sodium Benzoate treatment will have significant reductions in PANSS total score at week 12 compared to individuals taking placebo.

11.2 Endpoints

11.2.1 Primary

12 week treatment of 1000mg (500mg BD) Sodium Benzoate treatment improves the Positive and Negative Syndrome Scale (PANSS) total score compared to individuals taking placebo.

11.2.2 Secondary

12 week treatment of 1000mg (500mg BD) Sodium Benzoate treatment improves Positive and Negative Syndrome Scale (PANSS) subscales, Global Assessment of Function (GAF), Australian Quality of Life Scale (AQOL), The Activity and Participation Questionnaire (APQ6), Clinical Global Impression (CGI) and Hamilton Depression rating Scale-17items (HDRS) compared to individuals taking placebo.

11.2.3 Tertiary

Sera markers related to D-alanine, L-alanine, D-serine, L-serine, glycine and glutamate will change from baseline to endpoint in those on active treatment.

11.3 Sample Size and Power

The Lane *et al* study was based on n = 52 patients with chronic schizophrenia.[13] They reported a Cohen's effect size (d) of 1.53. Our patients (early psychosis) tend have slightly lower mean PANSS total scores (65.0), with a standard deviation of 14.3 units and it is anticipated that the effect size will be smaller in this group. With an alpha value of 0.05, and power of 0.8, we wish to be able to confidently detect a difference in mean PANSS Total of at least 7 units. This will require 66 participants per group (n = 132). Over a 12 week period we predict attrition of 15%. Thus, we will need to randomize approximately 160 subjects.

11.4 Statistical Analysis

All data will be analysed using SAS 9.4. We will compare demographic and clinical differences between the groups at baseline (Fisher exact test for nominal variables and Mann-Whitney test or independent sample t test for continuous variables). Efficacy will be assessed according to standard Intention to Treat (ITT) analytic procedures (i.e. for those who do not complete the 12 week study period, we will carry forward their last observation on the study outcomes). Mean changes in clinical assessment will be assessed using mixed-model repeated-measure (MMRM) methods with treatment, week, and treatment-week interaction as fixed effects and intercept as the only random effect; baseline value will be the covariant. The MMRM analyses will be performed using the SAS PROC Mixed procedure. P values will be based on 2-tailored tests with significance levels of 0.05.

12 Data Management

12.1 Documentation

A screening log will be utilized to track potential participants and also record the counts of individuals approached, consented, meeting inclusion/exclusion criteria, withdrawals, and completion (in keeping with standard CONSORT diagram requirements).

The Case Report Form (CRF) will comprise of the hard copy questionnaires, clinical assessments and measures. These de-identified data will be retained in a secure room, in a locked filing cabinet, at each site.

De-identified data from the CRFs will be entered into REDCap, which is a secure (encrypted to health service standard, housed on a server behind the University of Queensland firewall), web-based application for building and managing online surveys and databases. Delegated research assistants will be trained in, and responsible for, entering data into the database.

Upon completion and resolution of monitoring and data management queries, the clinical trial database will be closed. All data will be exported into SAS software to enable statistical analysis.

A copy of the PICF will be stored in a secure room in a locked filing cabinet separate from the CRFs.

12.2 Archiving

The Investigator, Project Manager or their delegate at each site will organise the retention of documentation relating to the study (source documents, informed consent forms, approvals) for a period of at least 15 years or the maximum time frame as determined by local regulations, whichever is the longest.

13 Monitoring and Quality Assurance

An independent Study Monitor will conduct study documentation review to monitor key features of the study prior to commencement, during and after study completion. These site visits will enable the Monitor to maintain current, personal knowledge of the study through review of the CRFs, comparison of CRF entries against the electronic data base (REDCap) and discuss the conduct of the study with the Investigator. The Monitor will be responsible for monitoring adherence to the approved study protocol, regulatory compliance including GCP and completion of the CRF. The organisation, supply of study materials and quality assurance of the clinical trial is the responsibility of the Investigator or its designee.

In order to ensure the accuracy of data, direct access to source documents by the representatives of both the Study Monitor and regulatory authorities will be available.

The Investigator will submit to the Reviewing HREC, annual (or more frequent if requested) reports of the study.

The study coordinator or designated delegate will monitor data entered at each site and be responsible for resolving data entry errors and discrepancies.

Data quality will be ensured by performing data entry checks for consistency between the CRF and the data entry into REDCap database. These checks will be performed during data entry so that discrepancies can be resolved immediately. A data manager will later perform additional checks for completeness and plausibility of data. Resultant queries will be raised and resolved electronically by the data manager and the study centre.

Each site will maintain a record of all personnel involved in the study including a Signature & Delegation Log which the Investigators will sign. In consultation with the lead site, each site will ensure that appropriate training is provided to study personnel, and that any new information of relevance to the performance of this study is forwarded to the staff involved in a timely manner.

13.1 Data Safety Monitoring Board

An independent Data Safety Monitoring Board (DSMB) will be established specifically to monitor safety data and study trends throughout the duration of the trial to determine if continuation of the trial is appropriate scientifically and ethically. The members of the DSMB serve in an independent capacity and will provide their expertise and recommendations to guide the clinical trial where required.

14 Investigator Responsibility

Except where the Coordinating Principal Investigator's signature is specifically required, it is understood that the term 'Investigator' as used in this Protocol and on the CRFs refers to the Coordinating Principal Investigator and the Principal Investigator or an appropriately qualified member of the staff that the Coordinating Principal Investigator designates to conduct the study. The Coordinating Principal Investigator is ultimately responsible for the conduct of all aspects of the study.

The study and its associated documents will be reviewed and approved by the appointed certified HREC and Research Governance (at all sites) before study start. A signed and dated letter that the ethics application has been approved by the appointed HREC and Research Governance Authority will be provided to the Sponsor before study initiation.

Prior to submission to appointed HREC and Research Governance, the investigator will sign the protocol signature page confirming his/her agreement to conduct the study in accordance with the protocol, GCP and other regulatory requirements locally applicable. All relevant data and records will be provided to study monitors, HREC and regulatory authorities as required. If an inspection of the clinical site is requested by a regulator, the investigator will inform the University of Queensland (Sponsor) immediately that this request has been received.

Each Investigator will comply with the local regulations regarding clinical trials and the Investigator responsibilities outlined in the ICH GCP guidelines.[1]

15 Study Report

The Investigator will submit at least annual study reports to the reviewing HREC, or more frequent if needed.

16 Administrative Procedures

16.1 Ethical Considerations

Information on side effects of the Investigational Product and reference formulations is summarised in the Investigator's Brochure. The monitoring and safety guidelines are outlined in the Monitoring Guidelines for the study. This study will be carried out according to the Declaration of Helsinki, the NHMRC National Statement on Ethical Conduct in Research Involving Humans (2007) and the Notes for Guidance on Good Clinical Practice as adopted by the Australian Therapeutic Goods Administration (2000) (CPMP/ICH/135/95) and the ICH GCP Guidelines.[1]

16.2 Ethical Review Committee

The National Ethics Application Form (NEAF) and associated documents will be submitted for approval to the appointed multi-site HREC and written approval obtained from both the appointed HREC and Governance Office, before volunteers are recruited and participants are enrolled. The Chief Investigator will submit the National Ethics Application Form and associated documents including Site Specific Applications from each site, to the appointed HREC and Research Governance. The Chief Investigator has overall responsibility to ensure all reports at each site are submitted in line with the appointed HREC reporting requirements.

16.3 Regulatory Authorities

The study will be notified under the Clinical Trial Notification (CTN) scheme. The University of Queensland (Sponsor) will submit the CTN forms from each participating site. The trial will also be listed on the Australian and New Zealand Clinical Trials Registry.

In agreeing to the provisions of the Protocol, these responsibilities are accepted by the Investigators.

16.4 Informed Consent

Our criteria will ensure that recruited participants will be sufficiently competent to consent and participate in the study or to refuse consent. Current research provides evidence that while psychotic symptoms may be present, these do not robustly predict an individual's functionality in daily life and capacity to make decisions, and whilst strongly correlated with cognitive impairment, do not reflect an enduring inability to understand information related to research participation.[42]

16.4.1 Adult participants (18-45 years inclusive)

Eligible adult participants (18-45 years) will be given a full explanation in lay terms, with a friend or family member present if desired, of the study aims, the discomfort, risks and benefits in taking part and a copy of the Participant Information Sheet Consent Form to review.

It will be pointed out to adult participants that they can withdraw from the study at any time without prejudice and will not affect their current care. The adult participants will have the opportunity to ask questions. A telephone number will be provided so that adult participants can call a research representative who will be able to respond to any questions they may have.

Each adult participant will acknowledge receipt of this information by giving written informed consent for participation in the study. The consent form will be signed and dated by a witness. A notation that written informed consent has been obtained will be made on the participant's Case

report Form (CRF). The original, completed consent forms will be retained by the Investigator and a copy will be provided by the research staff to the participants.

16.4.2 Young person participants (15<18years inclusive)

For eligible young person participants (15<18 years), a parent/ legal guardian will be present and give consent for the young person to participate. Consent will also be obtained from the young person. Both parent/legal guardian consent and child consent will be given to be eligible for participation.

It will be pointed out to the parent/legal guardian that consent can be withdrawn at any time without prejudice and withdrawal will not affect current treatment. The participant and parent/ legal guardian will have the opportunity to ask questions. A telephone number will be provided so that the participant and parent/legal guardian can call a research representative who will be able to respond to any questions they may have.

If the parent/legal guardian withdraws consent, regardless of the continuing young person's consent, then the young person will be withdrawn from the study.

If the young person's consent is withdrawn, regardless of the parent/legal guardian consent, then the young person's decision will be respected and they will be withdrawn from the study. This is to protect the young person's emotional wellbeing and continued ongoing routine care.

Each participant and parent/ legal guardian will acknowledge receipt of this information by giving written informed consent for participation in the study. The consent forms will be signed and dated by a witness. A notation that written informed consent has been obtained will be made in the participant's Case Report Form (CRF). The completed consent forms will be retained by the Investigator and a copy will be provided by the research staff to the participant.

16.5 Participant Reimbursement

Participants will be reimbursed for out of pocket expenses, inconvenience and time involved by the provision of prepaid gift cards (e.g. Coles-Myer, K-Mart etc). We will provide a $40 gift card at the end of week 2, and mid-way through the protocol (week 6) and on completion (week 12) (total reimbursement $120). If the study is terminated by the Investigator prior to completion, or a participant withdraws or is withdrawn from the study before completion, a pro-rata payment will be made at the discretion of the Investigator.

16.6 Emergency Contact with Investigators

All participants will be provided with a Participant Emergency Contact Card with contact details of whom to contact in the case of an emergency including unblinding.

16.7 Notification of Primary Care Physician and Treating Psychiatrist

With the consent of the participant, the Investigator will notify the primary care physician (provided that such a physician can be identified for the participant) and treating Psychiatrist of the participants' involvement in the study. A letter will be sent to the physician and treating Psychiatrist stating the nature of the study, treatments, expected benefits or adverse events. A copy will be retained by the study site for verification by the Study Monitor.

16.8 Investigator Indemnification

The clinical trial insurance will reimburse participants for costs of medical care that occur as a result of complications directly related to participation in this study. The Investigator and insurance company will be notified as soon as possible if this occurs or where a causal relationship cannot be excluded. All SAE's will be reported to the nominated insurance company.

The University of Queensland (Sponsor) will enter into a Clinical Trial Agreement with each of the five Hospital and Health Services (HHS's) involved in the study, based on the standard Medicines Australia format.

16.9 Intellectual Property (IP) and Licencing

The collection of data in this study is subject to Intellectual Property (IP) and Licencing agreements which will be documented in the Research Agreement.

16.10 Publication Policy

Results will be disseminated in peer reviewed publications and published in international journals. There will be an undertaking to seek journals that have open access policies. Our findings will also be summarised in several brochures, including one designed for feedback to participants and Hospital and Health Services (HHS's) who participate in the study. Only group data will be reported.

16.11 Protocol Amendments

Any amendments to the protocol will be submitted to the appointed HREC by the Chief Investigator for approval. Any approved amendments by the appointed HREC will be forwarded by the Chief Investigator for submission to each Research Governance Office.

No changes (amendments) to the Protocol will be implemented without prior approval from the Reviewing Ethics Committee. If a Protocol amendment requires changes to the Informed Consent Form, the revised Informed Consent Form, prepared by the Chief Investigator, will be approved by the Reviewing Ethics Committee and site governance officers.

Once the final Protocol has been issued and signed by the Chief Investigator and the authorised signatories, it will not be informally altered. All protocol amendments will pass through appropriate approval steps before being implemented. Any change to the protocol constitutes an amendment.

Where the amendment affects the ongoing suitability of the study at a participating site, Research Governance approval will also be sought. The Research Governance Office will determine the ongoing suitability based on the amendment submitted.

The Chief Investigator will submit the amendment to the appointed HREC for their approval; written approval will be obtained.   Completed and signed Protocol amendments will be circulated to all appointed site Investigators.

The original signed copy of amendments will be kept in the Study File with the original Protocol. Where an amendment to the Protocol substantially alters the study design or the potential risks to the participants, each participant's consent to continue participation will be obtained.

16.12 Version Control

Version control ensures that amendments to documents are tracked and verifiable and that the correct version of a document is in use according to the relevant ethical, regulatory or local approval.

All documents will be given a version number and date e.g. Version 1.0 15-Feb-15
Each amendment to a document will require a version number and date to be updated.

If this is a **significant change** e.g. change in the content of the document, then the version number will be increased by 1.0.

If it is a **minor change** e.g. contact details, then the number after the decimal point will be increased by 0.1.

16.13 Protocol Compliance

Should there be questions or consideration of deviation from the Protocol, clarification will be sought from the Study Monitor. Any participant treated in a manner that deviates from the Protocol, or who is admitted into the study but is not qualified according to the Protocol, will be ineligible for analysis.

If an emergency occurs that requires a departure from the Protocol, the nature and reasons for the Protocol violation/deviation will be recorded in the CRF and the Chief investigator will notify the Reviewing HREC and /or Governance Office as soon as possible.

Whilst the Chief Investigator has overall responsibility for the conduct of the study, the appointed site Investigators will have the responsibility to ensure all study personnel at their sites comply with GCP, National Statement on Ethical Conduct (2007), Australian Code for the Responsible Conduct of Research and local policies and procedures.

16.14 Archives: Retention of Study Records

All Case report Forms (CRF's) and study documentation will be kept by the Investigators for at least 15 years or the maximum time frame as determined by local regulations, whichever is the longest.

17 References

1. TGA. Note for Guidance on Good Clincal Practice (CMP/ICH/135/95) Annotated with TGA comments: Therapeutic Goods Administration, 2000.
2. Couture SM, Penn DL, Roberts DL. The functional significance of social cognition in schizophrenia: a review. *Schizophrenia bulletin* 2006; **32 Suppl 1**: S44-63.
3. Karagianis J, Novick D, Pecenak J, et al. Worldwide-Schizophrenia Outpatient Health Outcomes (W-SOHO): baseline characteristics of pan-regional observational data from more than 17,000 patients. *International journal of clinical practice* 2009; **63**(11): 1578-88.
4. Lieberman JA, Stroup TS, McEvoy JP, et al. Effectiveness of antipsychotic drugs in patients with chronic schizophrenia. *The New England journal of medicine* 2005; **353**(12): 1209-23.
5. Hashimoto K. Targeting of NMDA receptors in new treatments for schizophrenia. *Expert opinion on therapeutic targets* 2014; **18**(9): 1049-63.
6. Tsai GE, Lin PY. Strategies to enhance N-methyl-D-aspartate receptor-mediated neurotransmission in schizophrenia, a critical review and meta-analysis. *Current pharmaceutical design* 2010; **16**(5): 522-37.
7. Tsai GE, Yang P, Chang YC, Chong MY. D-alanine added to antipsychotics for the treatment of schizophrenia. *Biological psychiatry* 2006; **59**(3): 230-4.
8. Molla G, Sacchi S, Bernasconi M, Pilone MS, Fukui K, Polegioni L. Characterization of human D-amino acid oxidase. *FEBS letters* 2006; **580**(9): 2358-64.
9. Kawazoe T, Tsuge H, Imagawa T, Aki K, Kuramitsu S, Fukui K. Structural basis of D-DOPA oxidation by D-amino acid oxidase: alternative pathway for dopamine biosynthesis. *Biochemical and biophysical research communications* 2007; **355**(2): 385-91.
10. Ganote CE, Peterson DR, Carone FA. The nature of D-serine--induced nephrotoxicity. *The American journal of pathology* 1974; **77**(2): 269-82.
11. Maekawa M, Okamura T, Kasai N, Hori Y, Summer KH, Konno R. D-amino-acid oxidase is involved in D-serine-induced nephrotoxicity. *Chemical research in toxicology* 2005; **18**(11): 1678-82.
12. Ferraris D, Duvall B, Ko YS, et al. Synthesis and biological evaluation of D-amino acid oxidase inhibitors. *Journal of medicinal chemistry* 2008; **51**(12): 3357-9.
13. Lane HY, Lin CH, Green MF, et al. Add-on treatment of benzoate for schizophrenia: a randomized, double-blind, placebo-controlled trial of D-amino acid oxidase inhibitor. *JAMA psychiatry* 2013; **70**(12): 1267-75.
14. Lin CH, Chen PK, Chang YC, et al. Benzoate, a D-amino acid oxidase inhibitor, for the treatment of early-phase Alzheimer disease: a randomized, double-blind, placebo-controlled trial. *Biological psychiatry* 2014; **75**(9): 678-85.
15. Lai CH. Sodium benzoate, a D-amino acid oxidase inhibitor, increased volumes of thalamus, amygdala, and brainstem in a drug-naive patient with major depression. *The Journal of neuropsychiatry and clinical neurosciences* 2013; **25**(1): E50-1.
16. Lai CH, Lane HY, Tsai GE. Clinical and cerebral volumetric effects of sodium benzoate, a D-amino acid oxidase inhibitor, in a drug-naive patient with major depression. *Biological psychiatry* 2012; **71**(4): e9-e10.
17. Hou YC, Lai CH. A kind of D-amino acid oxidase inhibitor, sodium benzoate, might relieve panic symptoms in a first-episode, drug-naive panic-disorder patient. *The Journal of neuropsychiatry and clinical neurosciences* 2013; **25**(2): E7-8.
18. Rothschild DL, Jr. The Food Chemical News guide to the current status of food additivies and color additives. Washington, DC: Food and Chemical News; 1990.
19. Food and Agriculture Organization of the United Nations World Health Organization (FAO/WHO). Summary of evaluations perfomed by the Joint FAO/WHO Expert Committee on Food Addivites (JECFA). Washington, DC: International Life Sciences Institute; 1994.
20. Freedman BJ. Asthma induced by sulphur dioxide, benzoate and tartrazine contained in orange drinks. *Clinical allergy* 1977; **7**(5): 407-15.
21. World Health Organization. Safety evaluation of certain food additives. Geneva, 1999.

22. World Health Organization. Toxicological evaluation of certain food additives. Geneva, 1996.
23. Zengin N, Yuzbasioglu D, Unal F, Yilmaz S, Aksoy H. The evaluation of the genotoxicity of two food preservatives: sodium benzoate and potassium benzoate. *Food and chemical toxicology : an international journal published for the British Industrial Biological Research Association* 2011; **49**(4): 763-9.
24. Onodera H, Ogiu T, Matsuoka C, et al. Studies on effects of sodium benzoate on fetuses and offspring of Wistar rats. *Eisei Shikensho Hokoku* 1978; **96**: 47-55.
25. Batshaw ML, Brusilow SW. Evidence of lack of toxicity of sodium phenylacetate and sodium benzoate in treating urea cycle enzymopathies. *Journal of inherited metabolic disease* 1981; **4**(4): 231.
26. Batshaw ML, Monahan PS. Treatment of urea cycle disorders. *Enzyme* 1987; **38**(1-4): 242-50.
27. Green TP, Marchessault RP, Freese DK. Disposition of sodium benzoate in newborn infants with hyperammonemia. *The Journal of pediatrics* 1983; **102**(5): 785-90.
28. Stewart G, Sara G, Harris M, et al. A brief measure of vocational activity and community participation: development and reliability of the Activity and Participation Questionnaire. *The Australian and New Zealand journal of psychiatry* 2010; **44**(3): 258-66.
29. Hashimoto A, Nishikawa T, Oka T, Takahashi K, Hayashi T. Determination of free amino acid enantiomers in rat brain and serum by high-performance liquid chromatography after derivatization with N-tert.-butyloxycarbonyl-L-cysteine and o-phthaldialdehyde. *Journal of chromatography* 1992; **582**(1-2): 41-8.
30. Schizophrenia Working Group of the Psychiatric Genomics C. Biological insights from 108 schizophrenia-associated genetic loci. *Nature* 2014; **511**(7510): 421-7.
31. US FDA. GRAS (Generally Recongized As Safe) food ingredients: benzoic acid and sodium benzoate. Washington, DC: US Food and Drug Administration, 1972a.
32. US FDA. Evaluation of the health aspects of benzoic acid and sodium benzoate as food ingredients. Bethesda, MD.: US Food and Drug Administration, 1973.
33. Kubota K, Horai Y, Kushida K, Ishizaki T. Determination of benzoic acid and hippuric acid in human plasma and urine by high-performance liquid chromatography. *Journal of chromatography* 1988; **425**(1): 67-75.
34. Kubota K, Ishizaki T. Dose-dependent pharmacokinetics of benzoic acid following oral administration of sodium benzoate to humans. *European journal of clinical pharmacology* 1991; **41**(4): 363-8.
35. Feldmann RJ, Maibach HI. Absorption of some organic compounds through the skin in man. *The Journal of investigative dermatology* 1970; **54**(5): 399-404.
36. Feillet F, Leonard JV. Alternative pathway therapy for urea cycle disorders. *Journal of inherited metabolic disease* 1998; **21 Suppl 1**: 101-11.
37. Fujii T, Omori T, Tagucji T, Ogata M. Urinary excretion of hippuric acid after administration of sodium benzoate (biological monitoring 1). *Shokuhin Eiseigaku Zasshi (Journal of the Food Hygiene Society of Japan)* 1991; **32**(3): 177-82.
38. Bridges JW, French MR, Smith RL, Williams RT. The fate of benzoic acid in various species. *The Biochemical journal* 1970; **118**(1): 47-51.
39. World Health Organization. Benzoic Acid and Sodium Benzoate. Geneva: World Health, 2000.
40. World Health Organization. Recommened inn list 46 international nonproprietary names for pharmaceutical substances. *WHO Drug Information* 2001; **15**(3-4): 148-87.
41. Nettis E, Colanardi MC, Ferrannini A, Tursi A. Sodium benzoate-induced repeated episodes of acute urticaria/angio-oedema: randomized controlled trial. *The British journal of dermatology* 2004; **151**(4): 898-902.
42. Carpenter WT, Jr., Gold JM, Lahti AC, et al. Decisional capacity for informed consent in schizophrenia research. *Archives of general psychiatry* 2000; **57**(6): 533-8.
43. International Council of Beverages Associations, *ICBA Guidance Document to Mitigate the Potential for Benzene Formation in Beverages*. 2005-2006, UNESDA - Union of European Beverages Associations.
