# Supplementary material for: Effect of Sodium Benzoate vs Placebo Among Individuals With Early Psychosis: A Randomized Clinical Trial
Source: JAMA Netw Open. 2020 Nov 10;3(11):e2024335. doi: 10.1001/jamanetworkopen.2020.24335 (PMC7656289; doi:10.1001/jamanetworkopen.2020.24335)
Supplement: Supplement 2. — eTable 1. Comparisons of Medications Prescribed in the Benzoate and the Placebo Groups at Baseline eTable 2. Comparison of Total Number of Medications Prescribed in the Benzoate and the Placebo Groups at Baseline eTable 3. Per-Protocol Analysis: Clinical Measures of All Outcomes Over 12 Weeks Restricted to Those Who Did Not Miss 7 Days of Medication, Average Treatment Adherence of More Than 80%, and Completed the Trial eTable 4. End Point Results of Various Amino Acid Levels by Treatment Groups, Adjusting for Baseline Amino Acid Levels [file jamanetwopen-e2024335-s002.pdf]

Supplemental Online Content

Scott JG, Baker A, Lim CCW, et al. Effect of sodium benzoate vs placebo among individuals with early psychosis: a randomized clinical trial. *JAMA Netw Open*. 2020;3(11):e2024335. doi:10.1001/jamanetworkopen.2020.24335

eTable 1. Comparisons of Medications Prescribed in the Benzoate and the Placebo Groups at Baseline

eTable 2. Comparison of Total Number of Medications Prescribed in the Benzoate and the Placebo Groups at Baseline

eTable 3. Per-Protocol Analysis: Clinical Measures of All Outcomes Over 12 Weeks Restricted to Those Who Did Not Miss 7 Days of Medication, Average Treatment Adherence of More Than 80%, and Completed the Trial

eTable 4. End Point Results of Various Amino Acid Levels by Treatment Groups, Adjusting for Baseline Amino Acid Levels

This supplemental material has been provided by the authors to give readers additional information about their work.

© 2020 Scott JG et al. *JAMA Network Open.*

eTable 1. Comparisons of Medications Prescribed in the Benzoate and the Placebo Groups at Baseline

	Number of Participants		
**Type of Antipsychotic**	**Benzoate**	**Placebo**	**Total**
Aripiprazole	14	11	25
Amisulpride	2	2	4
Brexpiprazole	1	2	3
Clozapine	3	7	10
Haloperidol	1	0	1
Olanzapine	12	14	26
Lurasidone	3	2	5
Paliperidone	4	10	14
Quetiapine	7	4	11
Risperidone	7	10	17
**Type of mood stabilizer**	**Benzoate**	**Placebo**	**Total**
Lamotrigine	3	1	4
Lithium carbonate	4	5	9
Sodium valproate	3	1	4

© 2020 Scott JG et al. *JAMA Network Open.*

eTable 2. Comparison of Total Number of Medications Prescribed in the Benzoate and the Placebo Groups at Baseline

	Number of Participants		
**Number of antipsychotics**	**Benzoate**	**Placebo**	**Total**
0	3	1	4
1	38	36	74
2	8	13	21
**Number of mood stabilizers**			
0	39	43	82
1	9	7	16
2	1	0	1
**Antipsychotic only**	**39**	**43**	**82**
**Mood stabilizer only**	**3**	**1**	**4**
**Antipsychotic and mood stabilizer**	**7**	**6**	**13**

© 2020 Scott JG et al. *JAMA Network Open.*

Scale	Treatment period Least-Square Means (SE)						Placebo vs BZ		
							Difference in Score	Significant test	
	Week 2	Week 4	Week 6	Week 8	Week 10	Final visit	Mean (SE)	*t*	*p*-value
**<u>Primary measures</u>**									
**PANSS total score**									
Placebo	66.6 (1.8)	63.1 (1.8)	59.4 (1.8)	58.9 (1.8)	56.6 (1.8)	56.2 (1.8)	1.2 (2.6)	0.45	0.65
BZ	66.1 (1.9)	62.2 (1.9)	60.2 (1.9)	59.2 (1.9)	56.6 (1.9)	55.0 (1.9)			
**<u>Secondary measures</u>**									
**PANSS positive symptom subscale score**									
Placebo	15.4 (0.6)	14.4 (0.6)	14.1 (0.6)	13.8 (0.6)	13.9 (0.6)	13.0 (0.6)	0.9 (0.9)	1.01	0.31
BZ	15.4 (0.6)	14.1 (0.6)	13.2 (0.6)	12.8 (0.6)	12.3 (0.6)	12.1 (0.6)			
**PANSS positive symptom subscale score**									
Placebo	17.3 (0.7)	16.4 (0.7)	15.5 (0.7)	16.0 (0.7)	15.3 (0.7)	15.3 (0.7)	0.1 (1.0)	0.15	0.88
BZ	16.9 (0.7)	16.1 (0.7)	16.1 (0.7)	16.3 (0.7)	15.5 (0.7)	15.2 (0.7)			
**PANSS General psychopathology subscale score**									
Placebo	33.9 (1.0)	32.3 (1.0)	29.9 (1.0)	29.2 (1.0)	27.5 (1.0)	27.9 (1.0)	0.3 (1.4)	0.21	0.84
BZ	33.8 (1.0)	31.9 (1.0)	30.8 (1.0)	30.0 (1.0)	28.7 (1.0)	27.6 (1.0)			
**Clinical Global Impression (CGI)**									
Placebo	3.9 (0.1)	3.8 (0.1)	3.6 (0.1)	3.4 (0.1)	3.4 (0.1)	3.2 (0.1)	-0.4 (0.2)	-2.44	0.02
BZ	4.0 (0.1)	3.9 (0.1)	3.8 (0.1)	3.7 (0.1)	3.7 (0.1)	3.6 (0.1)			
**Global Assessment of Functioning (GAF)**									
Placebo	55.2 (1.2)	57.4 (1.2)	59.5 (1.2)	60.4 (1.2)	62.3 (1.2)	63.7 (1.2)	2.1 (1.8)	1.15	0.25
BZ	55.0 (1.3)	57.2 (1.3)	57.9 (1.3)	59.2 (1.3)	60.5 (1.3)	61.7 (1.3)			
**Hamilton Depression Rating Scale (HDRS)**									
Placebo	7.0 (0.7)	5.3 (0.7)	4.8 (0.7)	3.9 (0.7)	4.5 (0.7)	4.7 (0.7)	-0.6 (1.0)	-0.55	0.56
BZ	7.8 (0.7)	7.6 (0.7)	7.7 (0.7)	6.3 (0.7)	5.9 (0.7)	5.2 (0.7)			
**Assessment of Quality of Life (AQoL)**									
Placebo	83.0 (1.2)	85.4 (1.2)	87.7 (1.3)	87.9 (1.2)	88.6 (1.2)	88.9 (1.3)	2.5 (1.8)	1.39	0.16
BZ	83.3 (1.3)	84.4 (1.3)	84.2 (1.3)	85.7 (1.3)	86.4 (1.3)	86.3 (1.3)			

eTable 3. Per-Protocol Analysis: Clinical Measures of All Outcomes Over 12 Weeks Restricted to Those Who Did Not Miss 7 Days of Medication, Average Treatment Adherence of More Than 80%, and Completed the Trial

Abbreviations: PANSS, Positive and Negative Syndrome Scale; BZ, Sodiun benzoate, SE, Standard error

© 2020 Scott JG et al. *JAMA Network Open.*

	Placebo		Sodium Benzoate		H0: Baseline LS Mean = Endpoint LS Mean
	LS mean	SE	LS mean	SE	P-value
L_Glutamate	185.09	12.22	178.60	11.18	0.70
D_Glutamate	1.14	0.09	1.15	0.08	0.89
L_Serine	184.94	5.86	196.29	5.37	0.16
D_Serine	2.63	0.12	2.83	0.11	0.25
L_Alanine	508.65	18.48	536.65	16.93	0.27
D_Alanine	1.86	0.19	1.68	0.17	0.49
Glycine	414.51	15.45	430.01	14.15	0.46
Total Glutamate	186.25	12.25	179.74	11.21	0.70
Total Serine	187.58	5.90	199.11	5.41	0.16
Total Alanine	510.48	18.52	538.36	16.96	0.28
Ratio D_Glutamate/Total Glutamate	0.0063	0.0005	0.0070	0.0005	0.36
Ratio D_Serine/Total Serine	0.0145	0.0006	0.0143	0.0005	0.79
Ratio D_Alanine/Total Alanine	0.0036	0.0003	0.0032	0.0003	0.44

eTable 4. End Point Results of Various Amino Acid Levels by Treatment Groups, Adjusting for Baseline Amino Acid Levels*

Abbreviations: SE, Standard error; LS mean, Least-Square Means
*Results based on ANCOVA model, a blend of ANOVA and regression that evaluates whether the means of a dependent variable (i.e. endpoint blood serum measurement) are equal across levels of treatment group while controlling for the baseline blood serum measurement

© 2020 Scott JG et al. *JAMA Network Open.*
