# Supplementary material for: Effect of Sodium Benzoate vs Placebo Among Individuals With Early Psychosis: A Randomized Clinical Trial
Source: JAMA Netw Open. 2020 Nov 10;3(11):e2024335. doi: 10.1001/jamanetworkopen.2020.24335 (PMC7656289; doi:10.1001/jamanetworkopen.2020.24335)
Supplement: Supplement 3. — Data Sharing Statement [file jamanetwopen-e2024335-s003.pdf]

Data Sharing Statement

Scott. Effect of Sodium Benzoate vs Placebo Among Individuals With Early Psychosis. *JAMA Netw Open*. Published November 10, 2020. 10.1001/jamanetworkopen.2020.24335

Data
Data available: Yes
Data types: Deidentified participant data
How to access data: James.Scott@qimrberghofer.edu.au
When available: With publication

Supporting Documents
Document types: None

Additional Information
Who can access the data: Researchers whose proposed use of the data has been approved
Types of analyses: Related research (e.g. meta-analyses)
Mechanisms of data availability: With a signed data access agreement
Any additional restrictions: nil (note - full protocol previously published and uploaded with this submission)
